# BDNF-Hyaluronic Acid Hydrogel Promotes Neuronal Differentiation of Neural Stem Cells in Aβ-Induced Injury and 5×FAD Mice

**DOI:** 10.3390/biomedicines14061316

**Published:** 2026-06-10

**Authors:** Kangzhen Chen, Hehang Shi, Yuanyuan Bai, Shengbo Shi, Baoqing Gao, Hongmei Duan, Peng Hao, Wen Zhao, Yudan Gao, Zhaoyang Yang, Xiaoguang Li

**Affiliations:** Department of Neurobiology, School of Basic Medical Sciences, Capital Medical University, No. 10 Xitoutiao, You’anmen Wai, Fengtai District, Beijing 100069, China

**Keywords:** adult hippocampal neurogenesis, neural stem cells, brain-derived neurotrophic factor, hyaluronic acid hydrogel, neuronal differentiation, subgranular zone, subventricular zone

## Abstract

**Objectives**: Alzheimer’s disease (AD) is associated with impaired adult hippocampal neurogenesis (AHN). This study aimed to establish an in vitro model of Aβ_1–42_ oligomer-damaged neural stem cells (NSCs) and to employ the 5×FAD mouse model of AD in vivo, and to evaluate the therapeutic effects of brain-derived neurotrophic factor-loaded hyaluronic acid hydrogel (BDNF-HA gel) on AHN. **Methods**: In vitro, BDNF-HA gel was co-cultured with Aβ_1–42_ oligomer-impaired NSC spheres and evaluate NSC proliferation, migration, and differentiation. In vivo, BDNF-HA gel was infused intracerebroventricularly into 5×FAD mice. Using BrdU labeling, immunofluorescence, anterograde transsynaptic viral tracing, and behavioral tests, we assessed the effects of BDNF-HA gel on adult neurogenesis, newborn neuron integration into memory circuits, and cognitive function. **Results**: In vitro, BDNF-HA gel attenuated Aβ_1–42_-induced NSC apoptosis, restored proliferation and migration, promoted differentiation into neuroblasts, newborn neurons, and oligodendrocytes, and alleviated mitochondrial depolarization and loss of mitochondrial mass. In vivo, despite the absence of significant Aβ plaques reduction in 5×FAD mice, BDNF-HA gel markedly enhanced NSC proliferation and neurogenesis in the subventricular zone (SVZ) and subgranular zone (SGZ). Behavioral tests further revealed significant improvements in object recognition, spatial working memory, and spatial reference memory. **Conclusions**: BDNF-HA gel can effectively counteract the toxic microenvironment induced by Aβ oligomers, promoting NSC proliferation, migration, and differentiation into neurons. Without altering the Aβ burden, it significantly enhances adult neurogenesis and rescues cognitive deficits in AD mice.

## 1. Introduction

Alzheimer’s disease (AD) is a progressive neurodegenerative disorder characterized by memory impairment, extracellular deposition of insoluble amyloid-β plaques and intracellular neurofibrillary tangles formed by hyperphosphorylated tau protein [1,2]. These pathological processes ultimately cause serious neuronal damage and loss in the hippocampus, a structure essential for memory and cognition [3]. However, therapeutic strategies targeting Aβ or tau pathologies have thus far failed to confer meaningful cognitive benefits in patients. Numerous studies have demonstrated that adult neurogenesis is significantly reduced in both AD patients and animal models [4,5]. Moreover, impaired neurogenesis precedes Aβ plaque deposition and cognitive deficits in APP/PS1 and other AD transgenic mouse models, suggesting that this impairment may represent one of the early pathological features in AD progression [6]. Hence, new therapeutic strategies focusing on promoting neurogenesis to replenish damaged neuronal populations may represent a viable approach for AD [5,7,8,9].

In the adult mammalian brain, neural stem cells (NSCs) reside mainly in the subventricular zone (SVZ) of the lateral ventricles and the subgranular zone (SGZ) of the hippocampal dentate gyrus (DG). NSCs retain the ability to proliferate and differentiate into new neurons [10,11]. Adult hippocampal neurogenesis (AHN) refers to the continued production of new neurons from NSCs in the DG [12]. AHN declines significantly with aging, showing early-onset impairment and progressive deterioration during AD progression [12,13]. Notably, using doublecortin (DCX) as a marker for adult hippocampal neurogenesis, the number of DCX^+^ cells progressively declines in AD patients, with marked reductions observed at advanced Braak stages (IV-VI) relative to healthy controls [4]. Consequently, this compromised neurogenic capacity may contribute to the memory dysfunction and other cognitive deficits associated with AD pathophysiology.

Brain-derived neurotrophic factor (BDNF) is a member of the neurotrophin family and is abundant in the adult central nervous system. In addition, BDNF is critical for neuronal survival, synaptic plasticity, and cognitive functions [14,15]. Recent studies indicate that BDNF functions as a critical regulator of AHN, governing multiple stages of the neurogenic cascade [7,16,17,18,19]. This neurotrophic support is primarily mediated through activation of its high-affinity receptor TrkB, initiating downstream signaling pathways that promote neurogenic activity. Research indicates that serum BDNF levels in AD patients begin to decline during the early stage of the disease (the mild cognitive impairment phase) [20]. Therefore, elevating BDNF is promising for alleviating AD-associated deficits. However, the therapeutic efficacy of exogenous BDNF is largely hampered by its short half-life and vulnerability to degradation and inactivation. Thus, encapsulating BDNF in biodegradable carriers with sustained-release properties can overcome these limitations and enhance its biological activity. Hyaluronic acid (HA)-based hydrogels have emerged as promising candidates for this purpose, offering a unique combination of biocompatibility, tunable degradation, and controlled release kinetics [21].

In this study, we cultured Aβ-induced NSCs in the presence of BDNF hyaluronic acid hydrogels (BDNF-HA gel) in vitro. This in vitro model was used to mimic the toxic microenvironment of the AD brain, allowing us to examine how this sustained-release system affects NSC proliferation, migration, and differentiation into neuroblasts and newborn neurons. Subsequently, this sustained-release system was applied to 5×FAD transgenic mice to evaluate its capacity to restore adult neurogenesis in vivo. Our findings demonstrated that this BDNF-HA gel system could effectively promote the proliferation, migration and differentiation of Aβ_1–42_ oligomer-exposed NSCs into neuroblasts and newborn neurons. In addition, BDNF-HA gel also enhanced adult neurogenesis and ameliorated cognitive dysfunction in 5×FAD mice.

## 2. Materials and Methods

### 2.1. Neurosphere Isolation and Culture

Neurospheres were obtained from the hippocampus of postnatal Sprague-Dawley (SD) rats within the first 24 h of life. Briefly, newborn SD rats were disinfected with 75% ethanol. Under a stereomicroscope, the skull was carefully opened, and the meninges were gently peeled off with fine forceps to expose the brain. Bilateral hippocampal tissues were carefully isolated and immediately placed in ice-cold (4 °C) phosphate-buffered saline (PBS, pH 7.4) to maintain cell activity. Subsequently, the tissues were transferred to Dulbecco’s Modified Eagle Medium/Nutrient Mixture F-12 (DMEM/F-12) and rinsed three additional times to equilibrate them with the culture medium. The hippocampal tissues were minced into small fragments using sterile microscissors. The cell suspension was filtered through a 200-mesh sterile nylon filter to remove debris. The filtrate was collected into a 15 mL centrifuge tube and centrifuged at 1000 rpm for 5 min at room temperature. After carefully aspirating the supernatant, the cell pellet was resuspended in fresh complete medium. The complete medium was DMEM/F-12 medium containing 2% B27™ supplement (50×; Gibco, Life Technologies, Grand Island, NY, USA), 20 ng/mL epidermal growth factor (EGF; PeproTech, Rocky Hill, NJ, USA), 20 ng/mL basic fibroblast growth factor (FGF; PeproTech, Rocky Hill, NJ, USA), and 1% penicillin/streptomycin (10,000 U/mL, Gibco, Life Technologies, Grand Island, NY, USA). The cell suspension was transferred into sterile culture flasks and cultured in a humidified incubator with 5% (*v*/*v*) CO_2_ at 37 °C. Neurospheres were purified by passaging every three days, and neurospheres between passages 2 and 5 were subjected to further investigation.

### 2.2. Aβ Oligomers Preparation

Human amyloid β peptide (1–42) was obtained from Beyotime (Beyotime, Shanghai, China, P0023A) and prepared following the manufacturer’s instructions. The lyophilized peptide (1 mg) was first dissolved in 1 mL sterile saline to yield a 1 mg/mL stock solution, which was then aliquoted and stored at −20 °C. Before each experiment, the stock solution was thawed and diluted to the appropriate concentration using complete medium, followed by filtration through a 0.22 μm sterile filter to remove potential contaminants. The diluted peptide solution was then incubated at 37 °C for 24 h to promote oligomerization. Following centrifugation, the supernatant containing oligomeric Aβ was collected.

### 2.3. Construction of BDNF HA Gel

Hyaluronic acid powder (Sigma-Aldrich, St. Louis, MO, USA) was dissolved in sterile PBS (pH 7.2~7.4) with magnetic stirring at 4 °C to obtain a 0.5~5% (*w*/*v*) solution. Recombinant BDNF (Promega, Madison, WI, USA) was reconstituted in sterile PBS at 4 °C to a concentration of 50~200 μg/mL. The BDNF solution was slowly added to the HA solution at a final concentration of 50~200 ng/mL and gently mixed in an ice bath for 10~15 min to achieve uniform dispersion. The prepared solution was stored at 4 °C until use.

### 2.4. NSCs Differentiation

Neural stem cell spheres at passages 3–5 were seeded onto poly-L-lysine (PLL)–coated coverslips (10 μg/mL). After adhesion for 6 h, cells were randomly divided into three experimental groups and treated as follows: (1) Control group: cells were cultured in differentiation medium comprising DMEM/F-12 supplemented with 1% penicillin/streptomycin and 2% B27™ supplement (50×; Gibco, Life Technologies, Grand Island, NY, USA); (2) Aβ_1–42_ group: differentiation medium was supplemented with soluble Aβ_1–42_ oligomers at a final concentration of 10 μM and HA gel; (3) Aβ_1–42_ + BDNF-HA gel group: differentiation medium containing 10 μM Aβ_1–42_ oligomers was supplemented with BDNF-loaded hyaluronic acid gel. The differentiation process was monitored at four distinct time points: NSC proliferation was assessed at Day 7, neuroblast proliferation was evaluated at Day 14, newborn neuron proliferation and survival were examined at Day 21, and terminal neuronal differentiation and maturation were analyzed at Day 28.

### 2.5. TUNEL Assay for Apoptosis Detection

Apoptotic cell death within neurospheres was detected using a one-step terminal deoxynucleotidyl transferase dUTP nick-end labeling (TUNEL) assay kit (Beyotime, Shanghai, China, C1086). Briefly, neurospheres cultured on PLL-coated coverslips were rinsed three times with phosphate-buffered saline (PBS) and fixed with 4% paraformaldehyde for 15 min at room temperature. After fixation, samples were washed three times with PBS (5 min each) and permeabilized with 0.1% Triton X-100 (Solarbio, Beijing, China) in PBS for 30 min at room temperature. The TUNEL reaction mixture was prepared by mixing TdT enzyme and fluorescein-labeled dUTP reagent at a 1:9 ratio. Subsequently, 50 μL of TUNEL detection solution was applied to each coverslip, which was then covered with a sealing film to prevent evaporation and ensure uniform coverage, and incubated in a humidified chamber at 37 °C for 60 min in the dark. For nuclear counterstaining, cells were incubated with Hoechst 33342 (Life Technologies, Carlsbad, CA, USA) for 10 min at room temperature, followed by three additional washes with PBS (5 min each). Samples were mounted using antifade mounting medium (Servicebio, Wuhan, China) and examined under a fluorescence microscope.

### 2.6. Immunofluorescence Staining Analysis

Neurosphere differentiation was monitored by immunofluorescence across multiple time points. Neurospheres on PLL-coated coverslips were rinsed with PBS and fixed in 4% paraformaldehyde at room temperature for 15 min. Fixed samples were washed three times with PBS (5 min each), permeabilized with 0.1% Triton X-100 in PBS for 30 min, and blocked with 10% normal goat serum (NGS) at room temperature for 1 h. The following primary antibodies were diluted in PBS containing 5% NGS and incubated overnight at 4 °C: mouse anti-Sox2 (1:400, abcam, Cambridge, UK, ab79351), rabbit anti-Nestin (1:400, cell signaling technology, Danvers, MA, USA, 73349S), rabbit anti-DCX (1:400, abcam, Cambridge, UK, ab18723), mouse anti-βIII-tubulin (1:400, abcam, Cambridge, UK, ab78078), rabbit anti-beta-Amyloid (1:1000, cell signaling technology, Danvers, MA, USA, 8243S), rabbit anti-Olig2 (1:400, arigobio, Burlington, ON, Canada, ARG66828), chicken anti-GFAP (1:1000, abcam, Cambridge, UK, ab4674) and rat anti-Ki67 (1:300, Thermo Fisher Scientific, Waltham, MA, USA, 14-5698-82). After incubation, coverslips were washed three times with PBS for 5 min each. Corresponding Alexa Fluor–conjugated secondary antibodies (Alexa Fluor 488, 594, or 647; 1:400, Invitrogen, Carlsbad, CA, USA) were applied for 2 h at room temperature in the dark. Following secondary antibody incubation, samples were washed three times with PBS and counterstained with Hoechst 33342 (1:1000 in PBS) for 10 min to visualize cell nuclei. Coverslips were then mounted using antifade mounting medium (Servicebio, Wuhan, China, G1401). Fluorescence images were captured using a confocal laser scanning microscope (Leica STELLARIS 5, Leica Microsystems, Wetzlar, Germany).

### 2.7. BrdU Labeling Experiments

To trace the proliferation and differentiation of isolated NSC spheres, 5-Bromo-2-deoxyuridine (BrdU, B5002; Sigma-Aldrich Corporation, St. Louis, MO, USA) labeling experiments were performed. BrdU labeling medium was prepared by adding 1.5 mL of BrdU solution (1 mg/mL) to 48.5 mL of differentiation medium to achieve a final working concentration of 30 μg/mL. Neurospheres seeded on PLL-coated coverslips were cultured in this medium for 72 h with daily medium replenishment to maintain BrdU concentration. On day 4, BrdU-containing differentiation medium was replaced with standard differentiation medium corresponding to each experimental group.

For BrdU immunofluorescence cell staining, neurospheres were washed three times with PBS (5 min each) and fixed with 4% paraformaldehyde for 15 min at room temperature. Fixed samples were washed three times with PBS and permeabilized with 0.1% Triton X-100 in PBS for 30 min. To denature DNA and expose BrdU epitopes, cells were treated with 2N HCl for 10 min at 37 °C, followed by neutralization with 0.1 M sodium tetraborate (pH 8.5) for 15 min at room temperature. After three PBS washes, non-specific binding was blocked with 10% normal goat serum (NGS) in PBS for 1 h at room temperature. Primary antibody against BrdU (rat anti-BrdU, 1:400, abcam, Cambridge, UK, ab6326) was diluted in PBS containing 5% NGS and applied to coverslips overnight at 4 °C. After three PBS washes, Alexa Fluor 488-conjugated goat anti-rat secondary antibody (1:1000, Invitrogen, Grand Island, NY, USA) was applied for 2 h at room temperature protected from light. Samples were then counterstained with Topro (1:1000, Thermo Fisher Scientific, Waltham, MA, USA) for 10 min, washed three times with PBS, and mounted with antifade mounting medium (Servicebio, Wuhan, China, G1401).

To label proliferating NSCs, neuroblasts and neurons in the hippocampus of 5×FAD mice, BrdU labeling experiments were performed. BrdU was dissolved in sterile 0.9% saline at a working concentration of 10 mg/mL, filtered through a 0.22 μm membrane, and stored at 4 °C protected from light. Five-month-old mice received twice-daily intraperitoneal BrdU injections. For BrdU Immunofluorescence staining, 40 μm free-floating brain sections were washed three times with PBS for 5 min. To denature DNA and expose BrdU epitopes, sections were treated with 2N HCl for 15 min at 37 °C, followed by neutralization with 0.1 M sodium tetraborate (pH 8.5) for 15 min at room temperature. After permeabilization with 0.3% Triton X-100 in PBS for 30 min, non-specific binding was blocked with 10% NGS in PBS for 1 h at room temperature. Sections were then incubated with rat anti-BrdU primary antibody (rat anti-BrdU, 1:400, abcam, Cambridge, UK, ab6326) diluted in PBS containing 5% NGS for 48 h at 4 °C. Following primary antibody incubation, sections were washed three times with PBS and processed for secondary antibody detection.

### 2.8. Mitochondrial Membrane Potential Assessment

Mitochondrial membrane potential (MMP) was evaluated using the JC-1 assay kit (Beyotime, Shanghai, China, C2006) according to the manufacturer’s instructions. JC-1 staining solution was freshly prepared before each experiment by dissolving JC-1 (200×) in ultrapure water at a 1:160 ratio, followed by addition of JC-1 staining buffer (5×) to achieve a 1× working concentration. Neurospheres adhered to PLL-coated coverslips were gently rinsed twice with PBS and incubated with JC-1 staining working solution (1 mL per well for 24-well plates) at 37 °C for 30 min in a humidified incubator protected from light. Following staining, neurospheres were washed twice with ice-cold JC-1 staining buffer (1×) for 5 min each to remove unbound probe. For nuclear staining, cells were then incubated with Hoechst 33342 for 10 min at room temperature. Samples were immediately mounted with antifade mounting medium (Servicebio, Wuhan, China, G1401) and imaged using a confocal laser scanning microscope (Leica STELLARIS 5, Leica Microsystems, Wetzlar, Germany). JC-1 monomers (low ΔΨm) were detected using the FITC channel (excitation 488 nm, emission 525 ± 20 nm), while JC-1 aggregates (high ΔΨm) were visualized with the rhodamine channel (excitation 561 nm, emission 590 ± 20 nm). Mitochondrial depolarization was quantified by calculating the ratio of red to green fluorescence intensity using ImageJ software (version 1.54, National Institutes of Health, Bethesda, MD, USA).

### 2.9. Mito-Tracker Green Staining

To visualize mitochondrial morphology and distribution, Mito-Tracker Green staining (Beyotime, Shanghai, China, C1048) was performed on 4% paraformaldehyde-fixed samples. A 1 mM stock solution of Mito-Tracker Green was prepared in anhydrous DMSO and stored at −20 °C. The working solution was prepared by diluting the stock to a final concentration of 20 nM in PBS containing Ca^2+^ and Mg^2+^ and pre-warmed to 37 °C. Neurospheres were incubated with the working solution for 30 min at 37 °C in a humidified chamber protected from light. For nuclear staining, cells were then incubated with Hoechst 33342 for 10 min at room temperature. Samples were immediately mounted with antifade mounting medium (Servicebio, Wuhan, China, G1401) and imaged using a confocal laser scanning microscope (Leica STELLARIS 5, Leica Microsystems, Wetzlar, Germany).

### 2.10. Animals

5×FAD transgenic mice (C57BL/6J background), a well-established AD mouse line, express human APP and PSEN1 transgenes with five familial AD linked mutations: the Swedish (K670N/M671L), Florida (I716V), and London (V717I) mutations in APP, and the M146L and L286V mutations in PSEN1. The mice were obtained from Jackson Laboratory (Bar Harbor, ME, USA) and housed under standard specific-pathogen-free (SPF) conditions.

### 2.11. Intracerebroventricular Infusion of BDNF-HA Gel

Mice were anesthetized with isoflurane and secured in a stereotaxic apparatus. After disinfection, a midline incision was made along the sagittal suture to expose the skull, and the bregma was used as the reference point. A guide cannula was implanted into the lateral ventricle at the following coordinates: AP −0.45 mm, ML +1.1 mm, DV −1.8 mm relative to bregma. A burr hole was drilled at the target site, and the cannula was inserted. Three skull screws were placed around the cannula, and the assembly was fixed with dental cement. After surgery, the skin incision was sutured, and mice were placed on a heating pad until recovery. Before infusion, an internal cannula was connected to a microinjection pump via PE tubing. BDNF-HA gel was drawn into the internal cannula, and air bubbles were removed. After removing the dummy cap, the internal cannula was inserted, and 1.5 μL BDNF-HA gel was infused at a rate of 0.25 μL/min. The internal cannula was left in place for 3–5 min to prevent backflow, then slowly withdrawn, and the dummy cap was replaced.

### 2.12. Immunofluorescence Staining

After anesthesia, animals were perfused transcardially with 60 mL 0.9% saline followed by 4% paraformaldehyde (PFA) in PB. The extracted brain tissues were fixed overnight with 4% PFA, followed by 30% sucrose in PB for 3~5 days until they were fully submerged. Coronal brain slices at a thickness of 40 μm were stored in anti-freeze solution at −20 °C for further usage. For all staining, brain slices were pretreated as follows: they were washed three times for 5 min each in PBS, followed by 30 min permeabilization in 0.3% TritonX-100 in PBS (PBST), then 1 h of incubation in PBST containing 10% goat serum (Beyotime, Shanghai, China). Free-floating sections were incubated in primary antibody solution in PBST containing 5% goat serum at 4 °C for 48 h with the following primary antibodies: rat anti-Ki67 (1:400, Thermo Fisher Scientific, Waltham, MA, USA, 14-5698-82), rabbit anti-Aβ (1:1000, Cell Signaling Technology, Danvers, MA, USA, 8243T), rabbit anti-DCX (1:400, Cambridge, UK, abcam, ab18723), mouse anti-PSA-NCAM (1:400, 14-9118-82, Invitrogen), mouse anti-NeuN (1:400, Cambridge, UK, abcam, ab104224), rabbit anti-Parvalbumin (1:400, Cell Signaling Technology, Danvers, MA, USA, 80561), chicken anti-GFAP (1:1000, Cambridge, UK, abcam, ab4674) and mouse anti-Sox2 (1:400, Cambridge, UK, abcam, ab79351). After primary incubation, the free-floating sections went through 3 × 10 min wash steps in PBST. Then, sections were transferred to a secondary antibody solution in PBST for 2 h at room temperature in the dark with shaking. The secondary antibodies were Alexa Fluor-488/594-conjugated goat anti-rabbit IgG, Alexa Fluor-488/594-conjugated goat anti-rat IgG, Alexa Fluor-488/594-conjugated goat anti-mouse IgG, and Alexa Fluor-594/647-conjugated goat anti-chicken IgG (all 1:400, Jackson ImmunoResearch Laboratories, Inc., West Grove, PA, USA), respectively. Cell nuclei were counterstained with Hoechst 33342 (Life Technologies, Carlsbad, CA, USA) or Topro (1:1000, Invitrogen, Carlsbad, CA, USA, T3605) for 15 min. Sections were washed for 3 × 10 min in PBST. Finally, sections were mounted onto pathology-grade slides (CITOTEST, Haimen, China, 80302-0001) and covered with No.1.5 glass coverslips (CITOTEST, Haimen, China, 80341-4010).

For BrdU Immunofluorescence, frozen sections were washed three times with PBS and permeabilized with 0.3% Triton X-100 in PBS for 30 min. To denature DNA and expose BrdU epitopes, sections were treated with 2N HCl for 15 min at 37 °C, followed by neutralization with 0.1 M sodium tetraborate (pH 8.5) for 15 min at room temperature. After three PBS washes, non-specific binding was blocked with 10% normal goat serum (NGS) in PBS for 1 h at room temperature. Primary antibody against BrdU (rat anti-BrdU, 1:400, Cambridge, UK, abcam, ab6326) was diluted in PBS containing 5% NGS and applied to coverslips overnight at 4 °C.

### 2.13. Anterograde Transsynaptic Virus Injection

Recombinant HSV-1 strain H129 expressing EGFP (HSV-EGFP) was used for anterograde tracing. Virus was stored at −80 °C, thawed on ice, and used on the same day. Mice were anesthetized and fixed in a stereotaxic apparatus. A volume of 150 nL of virus was injected into the medial entorhinal cortex (AP: −4.24 mm, ML: +3.25 mm, DV: −3.5 mm relative to bregma) at 30 nL/min. The needle was left for 5 min before withdrawal. Bone defects were sealed with bone wax and dental cement. Post-surgery, mice were kept warm until recovery. Maximal viral infection occurred in the moribund state, characterized by loss of movement, absence of reflexes, and hypothermia. Mice were perfused when moribund symptoms appeared (typically 3–6 days post-injection).

### 2.14. Behavioral Experiments

Novel object recognition test: The test apparatus was a white acrylic box (40 cm × 40 cm × 30 cm) with a digital camera mounted above. During the habituation phase, mice were allowed to freely explore the empty box for 5 min. In the acquisition phase, two identical red wooden cubes (4.5 cm × 4.5 cm × 4.5 cm) were placed in the box, and mice were allowed to explore them for 5 min. Mice were then returned to their home cages for a 30 min retention interval. During the test phase, one of the cubes was replaced with a red wooden sphere (diameter 4.5 cm), and the time spent exploring the novel and familiar objects within 5 min was recorded. The recognition index was calculated as $\frac{exploration\;count\;for\;the\;novel\;object}{total\;exploration\;count\;for\;both\;objects}$ × 100%. The apparatus was cleaned with 75% ethanol between tests.

Y-maze test: The Y-maze consisted of three arms (30 cm long, 8 cm wide, 15 cm high) at 120° angles, with a camera mounted above. Mice were placed in the center of the maze and allowed to freely explore for 5 min. The sequence and total number of arm entries were recorded. Starting from the third entry, a correct alternation was defined as consecutive entries into three different arms. The spontaneous alternation rate was calculated as $\frac{number\;of\;correct\;alternations}{total\;arm\;entries-2}$ × 100%. The maze was cleaned with 75% ethanol between tests.

Barnes maze test: The Barnes maze comprised a circular platform (90 cm in diameter) elevated 50 cm above the floor, with 20 evenly distributed holes, one of which was the target hole connected to an escape box. The platform was surrounded by curtains with distinct visual cues placed in four directions. During the training phase (5 consecutive days, two trials per day, 3 min per trial), mice were placed in a start cylinder at the center of the platform. After 10–20 s, the cylinder was lifted, and mice were allowed to explore freely until they found the target hole. If a mouse failed to find the target hole within 3 min, it was guided to the hole. Mice were allowed to stay in the escape box for 1–2 min. During the test phase (24 h after the last training session, 90 s duration), the latency to first reach the target hole was recorded. The maze was cleaned with 75% ethanol between trials.

### 2.15. Statistical Analysis

Data were presented as mean ± standard deviation (SD). Measurements from individual experiments are shown on plots as datapoints wherever possible. GraphPad Prism 10.0 (GraphPad Software, San Diego, CA, USA) was used for statistical analyses and graphing of quantitative data. The Shapiro–Wilk test was used for data normality analysis, while Levene’s test was used to test for homogeneity of variance. Two groups were compared using two-tailed independent-samples Student’s *t*-test. Multiple group comparisons were performed by one-way analysis of variance (ANOVA), followed by Tukey’s post hoc test for pairwise comparisons. Outliers were detected using Grubbs’ test, and no data points were excluded. Post hoc power analyses were performed using G*Power 3.1.9.7 to confirm that the sample sizes were sufficient for the observed effect sizes. Details on the statistical tests used for each experiment are provided in the figure legends. A *p*-value of <0.05 was considered statistically significant.

## 3. Results

### 3.1. Cultured Neurospheres Express NSC Markers Nestin and Sox2

**Figure 1:**
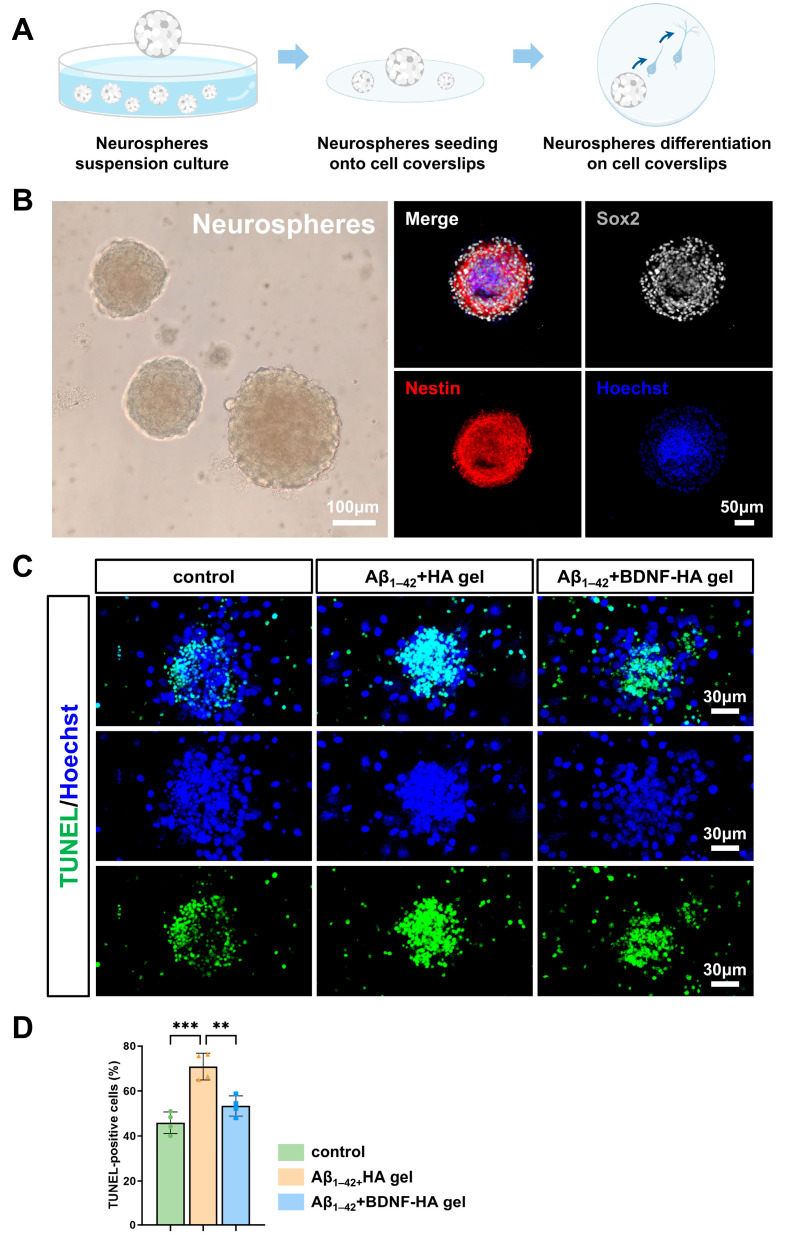
BDNF-HA gel attenuates neurosphere apoptosis induced by Aβ_1–42_ oligomers. (**A**) Schematic of neurosphere suspension culture and adherent differentiation. (**B**) Morphology of suspended neurospheres under light microscopy (scale bar: 100 μm) and Sox2 and Nestin immunofluorescence staining of NSCs after 5 h of adhesion (scale bar: 50 μm). (**C**) Immunofluorescence for TUNEL^+^ apoptotic cells (green), with Hoechst nuclear counterstaining (blue). Scale bar: 30 μm (**D**) Quantitative analysis of the percentage of TUNEL-positive cells. *n* = 4 independent experiments, one-way ANOVA, ** *p* < 0.01, *** *p* < 0.001. Data are presented as mean ± SD.

To verify whether the cultured suspended cell spheres possessed the properties of NSCs, we performed immunofluorescence staining for NSC-specific markers, such as Nestin and Sox2. Briefly, passaged cell spheres were seeded onto PLL-coated coverslips. After 5 h of incubation to allow neurosphere attachment, immunofluorescence staining was performed (Figure 1A). Two well-recognized markers were selected for this identification: Nestin and Sox2. Nestin, a class VI intermediate filament protein, is a classic marker for undifferentiated NSCs and neural progenitor cells. The high expression of Nestin is closely linked to the maintenance of NSCs’ immaturity and self-renewal ability. Sox2 is a member of the SRY-related high-mobility group (HMG)-box transcription factor family, which is critical for pluripotency and the maintenance of neural progenitor cell fate. Immunofluorescence analysis found that the adherent neurospheres exhibited robust co-expression of Nestin and Sox2 (Figure 1B). Collectively, these findings confirmed that the cultured suspended cell spheres were NSC spheres.

### 3.2. BDNF-HA Gel Attenuates Aβ Oligomer-Induced Apoptosis in NSC Spheres

To mimic the toxic effects of Aβ plaques on hippocampal NSCs in vivo, primary hippocampal neurospheres were treated with 10 μM Aβ_1–42_ oligomers. Apoptotic cells were assessed using the TUNEL assay. This method labels 3′-OH DNA ends with fluorescein-12-dUTP, marking cells in early apoptosis. Given that Aβ-induced apoptosis in NSCs mainly occurs during the early differentiation phase, TUNEL staining was conducted on neurospheres at day 3 of differentiation (Figure 1C). Compared to the control group, the number of TUNEL-positive apoptotic cells in Aβ_1–42_ oligomer-treated neurospheres was significantly increased (control: 45.85 ± 4.791% vs. Aβ_1–42_ + HA gel: 70.80 ± 5.920%; *p* < 0.001). This observation indicated that Aβ_1–42_ oligomers could directly induce apoptosis of hippocampus-derived NSC spheres, which is consistent with previous reports in AD mice, documenting the pro-apoptotic role of Aβ aggregates in the DG [22], and validates that the Aβ_1–42_-treated NSC spheres model could recapitulate the Aβ-induced neurotoxic microenvironment in vitro. Notably, BDNF-HA gel markedly reduced the number of TUNEL-positive cells in Aβ_1–42_-challenged NSC spheres (Aβ_1–42_ + HA gel: 70.80 ± 5.920% vs. Aβ_1–42_ + BDNF-HA gel: 53.34 ± 4.458%; *p* < 0.01) (Figure 1D), demonstrating that BDNF-HA gel exerts neuroprotective effects against Aβ_1–42_-mediated apoptotic damage.

### 3.3. BDNF-HA Gel Promotes Proliferation in Aβ_1–42_ Oligomer-Impaired NSCs

**Figure 2:**
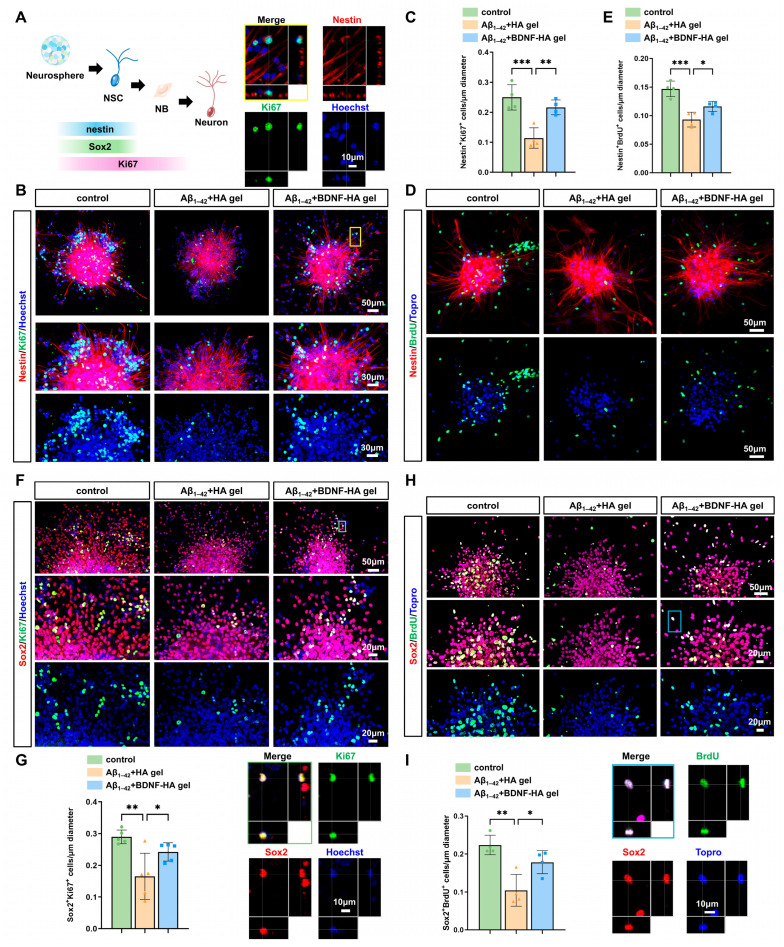
BDNF-HA gel promotes NSC proliferation under Aβ_1–42_ oligomer impairment. (**A**) Schematic illustration of the neurosphere differentiation process and stage-specific markers. (**B**) Immunofluorescence staining for Nestin^+^ NSCs (red) and Ki67^+^ proliferating cells (green), with Hoechst nuclear counterstaining (blue). The yellow box represents Nestin^+^Ki67^+^ cells, and above is an enlarged optical section of the cells within the yellow box. Scale bar: 50 μm (top row) and 30 μm (middle and bottom rows). (**C**) Quantification of Nestin^+^ Ki67^+^ cells. *n* = 4 independent experiments, one-way ANOVA, ** *p* < 0.01, *** *p* < 0.001. Data are presented as mean ± SD. (**D**) Immunofluorescence staining for Nestin^+^ NSCs (red) and BrdU^+^ proliferating cells (green), with Topro nuclear counterstaining (blue). Scale bar: 50 μm. (**E**) Quantification of Nestin^+^ BrdU^+^ cells. *n* = 4 independent experiments, one-way ANOVA, * *p* < 0.05, *** *p* < 0.001. Data are presented as mean ± SD. (**F**) Immunofluorescence staining for Sox2^+^ NSCs (red) and Ki67^+^ proliferating cells (green), with Hoechst nuclear counterstaining (blue). The green box represents Sox2^+^Ki67^+^ cells, and below is an enlarged optical section of the cells within the green box. Scale bar: 50 μm (top row) and 20 μm (middle and bottom rows). (**G**) Quantification of Sox2^+^ Ki67^+^ cells. *n* = 5 independent experiments, one-way ANOVA, * *p* < 0.05, ** *p* < 0.01. Data are presented as mean ± SD. (**H**) Immunofluorescence staining for Sox2^+^ NSCs (red) and BrdU^+^ proliferating cells (green), with Topro nuclear counterstaining (blue). The blue box represents Sox2^+^BrdU^+^ cells, and below is an enlarged optical section of the cells within the blue box. Scale bar: 50 μm (top row) and 20 μm (middle and bottom rows). (**I**) Quantification of Sox2^+^ BrdU^+^ cells. *n* = 4 independent experiments, one-way ANOVA, * *p* < 0.05, ** *p* < 0.01. Data are presented as mean ± SD.

AHN occurs in the SGZ of the hippocampal DG. This dynamic process spans stem cell division, neuronal or glial differentiation, and eventual circuit integration. The AHN impairment is a hallmark pathological feature of AD. To investigate the effect of BDNF-HA gel on neurogenesis in Aβ_1–42_ oligomer-damaged NSCs, the NSCs proliferation was evaluated. Neurospheres at day 7 of differentiation were subjected to immunofluorescence staining for the NSC marker Nestin and the proliferation marker Ki67 (Figure 2A). The results revealed that the number of Nestin^+^Ki67^+^ double-positive cells was significantly reduced in neurospheres treated with Aβ_1–42_ oligomers compared with that in the control group, indicating that Aβ_1–42_ oligomers suppressed the proliferation of hippocampus-derived NSCs (control: 0.249 ± 0.042 vs. Aβ_1–42_ + HA gel: 0.114 ± 0.033; *p* < 0.001). In contrast, the addition of BDNF-HA gel to Aβ_1–42_-treated neurospheres effectively rescued this deficit, leading to a significant increase in the population of proliferating NSCs (Aβ_1–42_ + HA gel: 0.114 ± 0.033 vs. Aβ_1–42_ + BDNF-HA gel: 0.216 ± 0.024; *p* < 0.01) (Figure 2B,C). Additionally, we combined Nestin and BrdU staining to detect proliferating NSCs (Figure 2D), and the results were consistent with those described above. Compared with the Aβ_1–42_ + HA gel group, the BDNF-HA gel significantly increased the number of Nestin^+^ BrdU^+^ cells (Figure 2E). These findings collectively demonstrate that BDNF-HA gel exerts a potent pro-proliferative effect on Aβ_1–42_ oligomer-damaged hippocampal NSCs.

To further validate these findings, we additionally performed immunofluorescence staining for another NSC marker, Sox2, combined with Ki67 to co-label proliferating NSCs (Figure 2F). The results of Sox2^+^Ki67^+^ co-staining were consistent with those of Nestin^+^Ki67^+^ staining. Compared with the control group, the number of Sox2^+^Ki67^+^ proliferating NSCs in Aβ_1–42_ oligomer-damaged NSC spheres was significantly decreased, corroborating the inhibitory effect of Aβ_1–42_ oligomers on NSC proliferation (control: 0.289 ± 0.021 vs. Aβ_1–42_ + HA gel: 0.164 ± 0.073; *p* < 0.01) (Figure 2G). Importantly, co-administration of the BDNF-HA gel to the Aβ_1–42_ injury model markedly increased the number of Sox2^+^Ki67^+^ cells relative to the Aβ_1–42_-alone group (Aβ_1–42_ + HA gel: 0.164 ± 0.073 vs. Aβ_1–42_ + BDNF-HA gel: 0.242 ± 0.028; *p* < 0.05) (Figure 2G). Additionally, we combined Sox2 and BrdU staining to detect proliferating NSCs (Figure 2H), and the results were consistent with those described above. Compared with the Aβ_1–42_ group, the BDNF-HA gel significantly increased the number of Sox2^+^ BrdU^+^ cells (Figure 2I). These findings establish BDNF-HA gel as a rescue strategy for Aβ-impaired NSC proliferation, thereby promoting neurogenesis.

### 3.4. BDNF-HA Gel Promotes Migration in Aβ_1–42_ Oligomer-Impaired NSCs

**Figure 3:**
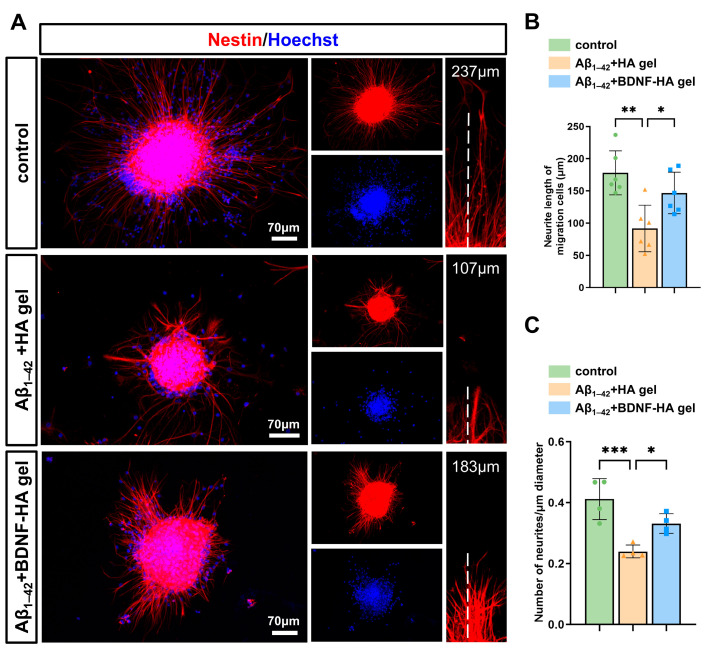
BDNF-HA gel promotes NSC migration under Aβ_1–42_ oligomer impairment. (**A**) Immunofluorescence staining for Nestin^+^ NSCs (red), with Hoechst nuclear counterstaining (blue). The white dashed line indicates the length of the neurite. Scale bar: 70 μm. (**B**) Quantitative analysis of the neurite length of migrating cells. *n* = 6 independent experiments, one-way ANOVA, * *p* < 0.05, ** *p* < 0.01. Data are presented as mean ± SD. (**C**) Quantitative analysis of neurite number. *n* = 4 independent experiments, one-way ANOVA, * *p* < 0.05, *** *p* < 0.001. Data are presented as mean ± SD.

NSC migration and neurite outgrowth are essential prerequisites for neurogenesis. NSC spheres plated on PLL-coated coverslips showed time-dependent migration. NSCs gradually migrated outward radially from the spheres, with extending neurites attaching to the coverslip surface. To quantitatively evaluate the migration capacity and developmental potential of NSCs in each group, we measured two key indicators: the length of migrated NSC neurites and the total number of neurites per NSC sphere (Figure 3A). The average neurite length of migrated NSCs in the control group was 178.2 μm, which was significantly longer than that in the Aβ_1–42_ oligomer-treated group (91.83 μm, *p* < 0.01). This result indicates that Aβ_1–42_ oligomers exert a potent inhibitory effect on NSC migration. Notably, co-treatment with BDNF-HA gel reversed this inhibitory effect. The average neurite length of NSCs in the Aβ_1–42_ + BDNF-HA gel group was 146.8 μm, which was significantly increased compared with the Aβ_1–42_ + HA gel group (*p* < 0.05), though not fully restored to the level of the control group (Figure 3B). In addition, neurite number dropped sharply with Aβ_1–42_ + HA gel exposure (*p* < 0.001). Co-treatment with BDNF-HA gel significantly attenuated this reduction (*p* < 0.05) (Figure 3C). Taken together, these results reveal a significant disruption in NSC migratory function caused by Aβ_1–42_ oligomers, characterized by a notable reduction in both neurite length and number. Notably, this detrimental effect was largely reversed by the application of the BDNF-HA gel.

### 3.5. BDNF-HA Gel Rescues Neuroblast Generation from Aβ_1–42_-Impaired NSCs

**Figure 4:**
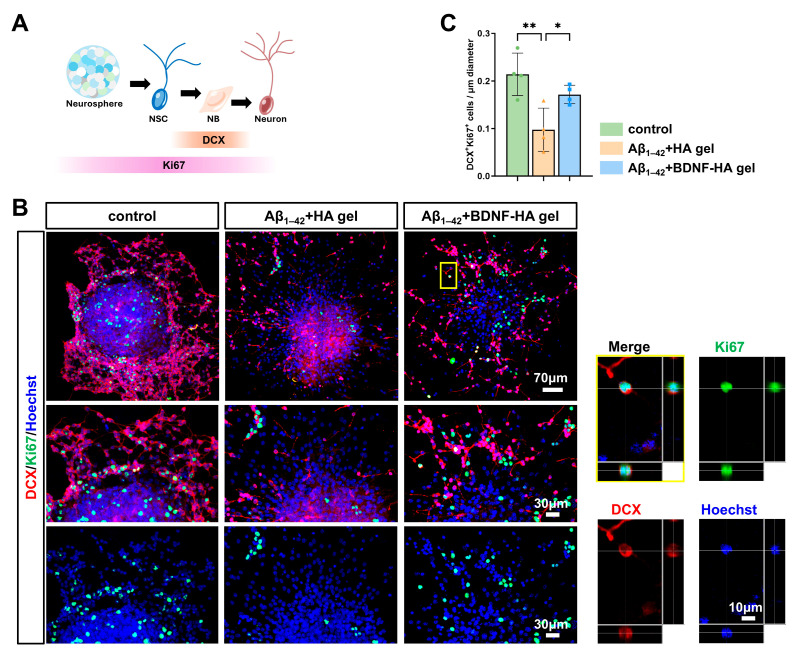
BDNF-HA gel promotes neuroblast generation under Aβ_1–42_ oligomer impairment. (**A**) Schematic illustration of the neurosphere differentiation process and stage-specific markers. (**B**) Immunofluorescence staining for DCX^+^ neuroblasts (red) and Ki67^+^ proliferating cells (green), with Hoechst nuclear counterstaining (blue). The yellow box represents DCX^+^Ki67^+^ cells, and on the right is an enlarged optical section of the cells within the yellow box. Scale bar: 70 μm (top row) and 30 μm (middle and bottom rows). (**C**) Quantification of DCX^+^ Ki67^+^ cells. *n* = 4 independent experiments, one-way ANOVA, * *p* < 0.05, ** *p* < 0.01. Data are presented as mean ± SD.

Neuroblasts, as transient intermediate progenitors, originate from differentiation of NSCs, undergo migration and further maturation, and finally differentiate into neurons. These cells express Doublecortin (DCX), a microtubule-associated protein essential for neuronal migration and differentiation. Day 14 spheres received DCX/Ki67 double staining to probe the effect of BDNF-HA gel on neuronal fate in Aβ_1–42_-exposed NSCs (Figure 4A). This time window was deliberately selected to ensure that NSCs had sufficient time to differentiate into neuroblasts. Quantification revealed that Aβ_1–42_ oligomer treatment dramatically reduced the number of DCX^+^Ki67^+^ double-positive cells compared to control (*p* < 0.01), demonstrating that Aβ pathology impairs not only the proliferation of NSCs but also the expansion of neuronal progenitors. This finding aligns with previous clinical evidence demonstrating that DCX^+^PCNA^+^ neuroblasts are significantly diminished in the hippocampus of MCI patients [13]. Notably, the addition of the BDNF-HA gel to Aβ_1–42_-treated cultures effectively reversed this decline. The number of DCX^+^Ki67^+^ cells in the Aβ_1–42_ + BDNF-HA gel group was significantly higher than in the Aβ_1–42_ + HA gel group (*p* < 0.05) (Figure 4B,C). These results confirm that BDNF-HA gel can rescue neurogenesis under Aβ-toxic conditions by restoring the proliferation of neuroblasts.

### 3.6. BDNF-HA Gel Preferentially Rescues Neuronal and Oligodendroglial Differentiation from Aβ_1–42_-Impaired NSCs

**Figure 5:**
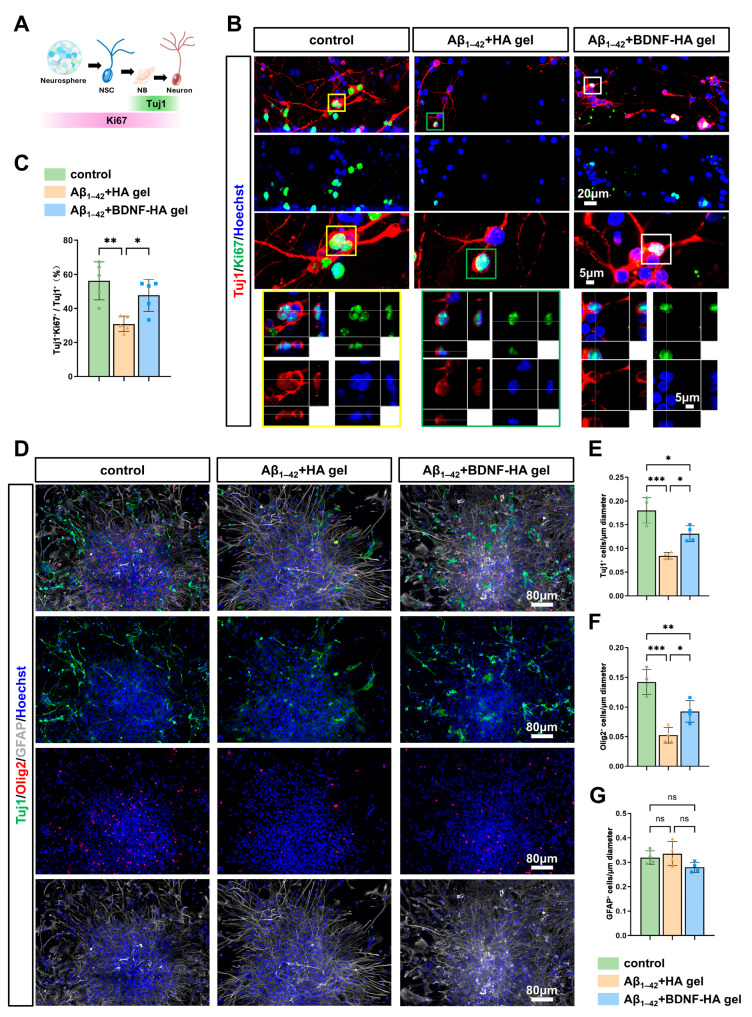
BDNF-HA gel promotes neuronal and oligodendroglial differentiation under Aβ_1–42_ oligomer impairment. (**A**) Schematic illustration of the neurosphere differentiation process and stage-specific markers. (**B**) Immunofluorescence staining for Tuj1^+^ newborn neurons (red) and Ki67^+^ proliferating cells (green), with Hoechst nuclear counterstaining (blue). The yellow, green, and white boxes represent Tuj1^+^Ki67^+^ cells in the control group, the Aβ_1–42_+HA gel group, and the Aβ_1–42_+BDNF-HA gel group, respectively. Below are the optical section images of the cells within the boxes. Scale bar: 20 μm (top and middle rows) and 5 μm (bottom row). (**C**) Quantification of the percentage of Tuj1^+^ Ki67^+^ cells among total Tuj1^+^ cells. *n* = 5 independent experiments, one-way ANOVA, * *p* < 0.05, ** *p* < 0.01. Data are presented as mean ± SD. (**D**) Immunofluorescence staining for Tuj1^+^ newborn neurons (green), Olig2^+^ oligodendrocytes (red) and GFAP^+^ astrocytes (white), with Hoechst nuclear counterstaining (blue). Scale bar: 80 μm. (**E**) Quantification of Tuj1^+^ cells. *n* = 4 independent experiments, one-way ANOVA, * *p* < 0.05, *** *p* < 0.001. Data are presented as mean ± SD. (**F**) Quantification of Olig2^+^ cells. *n* = 4 independent experiments, one-way ANOVA, * *p* < 0.05, ** *p* < 0.01, *** *p* < 0.001. Data are presented as mean ± SD. (**G**) Quantification of GFAP^+^ cells. *n* = 4 independent experiments, one-way ANOVA. Data are presented as mean ± SD. There was no statistically significant difference among groups.

To evaluate the effect of BDNF-HA gel on the generation of newborn neurons following Aβ_1–42_-induced impairment, immunofluorescence co-staining for βIII-Tubulin (Tuj1) and the proliferation marker Ki67 was performed on neurospheres at day 21 of differentiation (Figure 5A). Consistent with the impairment observed at earlier progenitor stages, treatment with Aβ_1–42_ oligomers significantly reduced the population of Tuj1^+^/Ki67^+^ newborn neurons compared to the control group (control: 56.19 ± 11.170% vs. Aβ_1–42_ + HA gel: 30.92 ± 4.570%; *p* < 0.01). These findings indicate that Aβ toxicity not only inhibits the proliferation of neural stem and progenitor cells, but also markedly reduces the generation of newborn neurons. Notably, this deficit was effectively rescued by the BDNF-HA gel. Co-treatment with BDNF-HA gel markedly increased the population of Tuj1^+^Ki67^+^ newborn neurons relative to the Aβ_1–42_ group (*p* < 0.05) (Figure 5B,C). These results suggest that BDNF-HA gel effectively promotes neuronal production from Aβ_1–42_-injured NSCs.

Besides neurogenesis, cultured neurospheres can also differentiate into astrocytes and oligodendrocytes, giving rise to all three major neural cell lineages. To explore whether BDNF-HA gel affects the multipotent differentiation of NSCs, we performed immunofluorescence staining on neurospheres at differentiation day 28 to detect three major neural lineage markers: Tuj1 for immature neurons, GFAP for astrocytes, and Olig2 for oligodendrocyte lineage cells (Figure 5D). Compared with the control group, the Aβ_1–42_ oligomer-treated group exhibited a marked reduction in both Tuj1^+^ newborn neurons and Olig2^+^ oligodendrocytes (*p* < 0.001). BDNF-HA gel co-treatment recovered the numbers of Tuj1^+^ and Olig2^+^ cells despite Aβ_1–42_ exposure, enabling neuronal and oligodendroglial differentiation (*p* < 0.05) (Figure 5E,F). These results suggest that BDNF-HA gel effectively supports the differentiation of NSCs toward neuronal and oligodendroglial lineages under Aβ-induced toxic conditions. Moreover, the number of GFAP-positive astrocytes showed no significant difference among the three groups (Figure 5G). Taken together, these results indicate that BDNF-HA gel preferentially protects neuronal and oligodendrocyte lineages from Aβ_1–42_-impaired NSCs.

### 3.7. BDNF-HA Gel Mitigates Mitochondrial Dysfunction Under Aβ_1–42_ Oligomer Impairment

**Figure 6:**
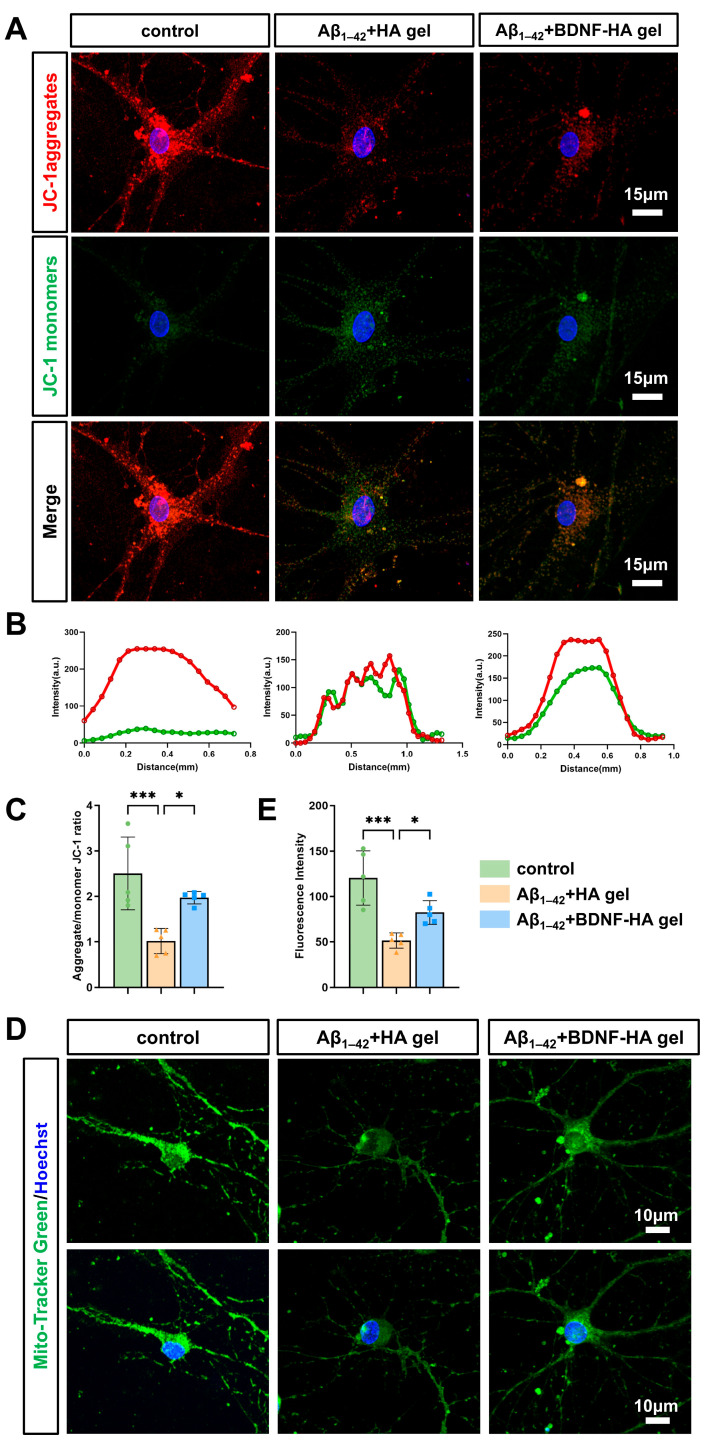
BDNF-HA gel mitigates mitochondrial dysfunction under Aβ_1–42_ oligomer impairment. (**A**) Representative images showing JC-1 staining for mitochondrial membrane potential. JC-1 aggregates (red) indicate polarized mitochondria with high membrane potential, while JC-1 monomers (green) indicate depolarized mitochondria. Hoechst 33342 (blue) was used for nuclear counterstaining. Scale bar: 15 μm. (**B**) Fluorescence intensity profiles along the indicated line scans showing the distribution of JC-1 aggregates (red) and monomers (green). (**C**) Quantification of the aggregate-to-monomer JC-1 ratio as an indicator of mitochondrial membrane potential. *n* = 5 independent experiments, one-way ANOVA, * *p* < 0.05, *** *p* < 0.001. Data are presented as mean ± SD. (**D**) Representative images stained with Mito-Tracker Green (green) for mitochondrial morphology and Hoechst 33342 (blue) for nuclei. Scale bar: 10 μm. (**E**) Quantification of mitochondrial fluorescence intensity. *n* = 5 independent experiments, one-way ANOVA, * *p* < 0.05, *** *p* < 0.001. Data are presented as mean ± SD.

Neurogenesis is an energy-intensive process that largely depends on mitochondrial function to support cell proliferation, differentiation, and neuronal maturation. Impaired mitochondrial membrane potential (MMP) and reduced mitochondrial mass are closely associated with Aβ-induced neurogenic defects in AD. To investigate whether BDNF-HA gel preserves mitochondrial function against Aβ_1–42_-induced injury, we assessed MMP with the JC-1 fluorescent probe and measured mitochondrial mass by Mito-Tracker Green staining. High membrane potential drives JC-1 into red-fluorescent aggregates. Loss of potential shifts the dye to its green monomeric form (Figure 6A,B). The red/green ratio dropped sharply with Aβ_1–42_ exposure, indicating mitochondrial depolarization (*p* < 0.001 vs. control). Co-treatment with BDNF-HA hydrogel rescued this loss (*p* < 0.05 vs. Aβ_1–42_ + HA gel) (Figure 6C). To further evaluate mitochondrial mass, Mito-Tracker Green staining was performed (Figure 6D). Aβ_1–42_ exposure decreased mitochondrial mass (*p* < 0.001), whereas BDNF-HA gel partially preserved mitochondrial content (*p* < 0.05 vs. Aβ_1–42_ + HA gel) (Figure 6E). These findings indicate that BDNF-HA hydrogel mitigates Aβ_1–42_-induced mitochondrial depolarization and loss. BDNF-HA gel promotes neurogenesis in part by restoring mitochondrial health.

### 3.8. Neurogenic Effects of BDNF-HA Gel Are Independent of Aβ Reduction

**Figure 7:**
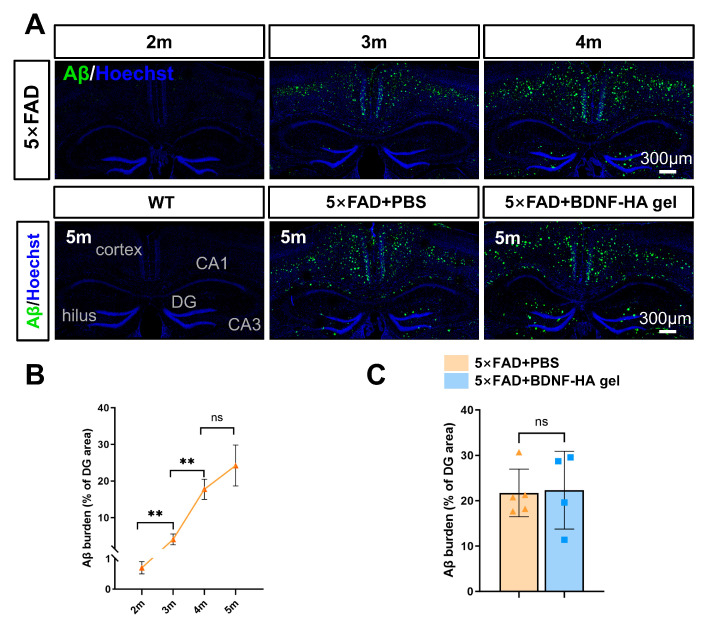
BDNF-HA gel treatment does not reduce Aβ plaque burden in 5×FAD mice. (**A**) Immunofluorescence images showing Aβ deposition (green) in the hippocampus of 5×FAD mice at 2, 3, and 4 months of age (top row), and comparison of WT, 5×FAD + PBS, and 5×FAD + BDNF-HA gel groups at 5 months (bottom row). Hoechst (blue) was used for nuclear counterstaining. (**B**) Quantification of Aβ burden (% of DG area) in 5×FAD mice showing age-dependent accumulation of Aβ plaques from 2 to 5 months. *n* = 3 independent experiments, Student’s *t*-test, ** *p* < 0.01. Data are presented as mean ± SD. No significant difference was observed between 4 m and 5 m. (**C**) Quantification of Aβ burden (% of DG area) in 5×FAD mice treated with PBS or BDNF-HA gel at 5 months. *n* = 4–5 independent experiments, Student’s *t*-test. No significant difference was observed between the two groups.

To validate these findings in vivo, we employed 5×FAD mice, which exhibit progressive Aβ accumulation and neurogenesis impairment. A time-course analysis of Aβ deposition between 2 and 5 months of age revealed a clear age-dependent progression of pathology. Starting at 2 months, only minimal plaques were detectable in the hippocampus, with sparse deposition in the cortex. By 3 months, modest plaque formation emerged in the hippocampus, accompanied by a prominent accumulation in the cortex. At 4 months, both regions exhibited substantial Aβ burden, which further intensified throughout the hippocampus and cortex by 5 months (Figure 7A,B). To investigate whether the BDNF-HA gel influences Aβ pathology in vivo, 5×FAD mice were treated with BDNF-HA gel from 3 months (initial Aβ deposition) to 5 months of age. BDNF-HA gel treatment did not significantly alter Aβ deposition in hippocampal DG compared with the 5×FAD + PBS group (Figure 7C). These results confirm that BDNF-HA gel does not modulate Aβ deposition in 5×FAD mice. Aβ deposition is one pathological outcome. Cognitive function depends more on neuronal network integrity, and its causal relationship with Aβ is not straightforward. Even with Aβ present, cognitive recovery may be achieved through the maintenance of neuronal function and synaptic plasticity.

### 3.9. BDNF-HA Gel Promotes NSCs Proliferation in the SVZ of 5×FAD Mice

**Figure 8:**
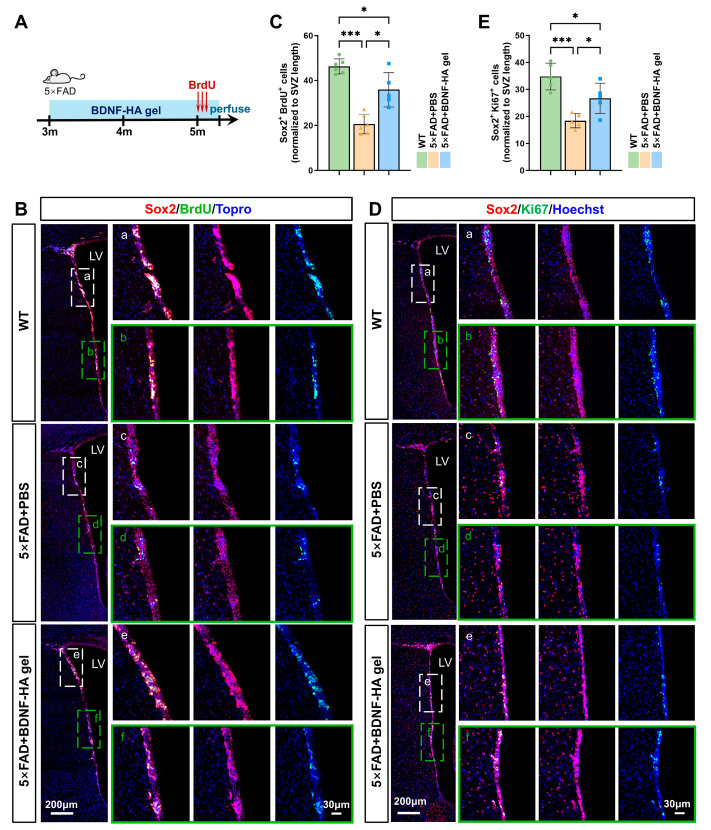
BDNF-HA gel promotes NSC proliferation in the SVZ of 5×FAD mice. (**A**) Experimental timeline showing BDNF-HA gel implantation in 3-month-old mice, BrdU administration at 5 months of age, and perfusion 24 h after the last BrdU injection. (**B**) Immunofluorescence staining for Sox2^+^ NSCs (red) and BrdU^+^ proliferating cells (green), with Topro nuclear counterstaining (blue). a/c/e represent the dorsolateral wall of the lateral ventricle (LV), while b/d/f represent the ventrolateral wall of the LV. Scale bars: 200 μm (low magnification), 30 μm (high magnification). (**C**) Quantification of Sox2^+^BrdU^+^ cells normalized to SVZ length. *n* = 5~6 independent experiments, one-way ANOVA, * *p* < 0.05, *** *p* < 0.001. Data are presented as mean ± SD. (**D**) Immunofluorescence staining for Sox2^+^ NSCs (red) and Ki67^+^ proliferating cells (green), with Hoechst nuclear counterstaining (blue). a/c/e represent the dorsolateral wall of the LV, while b/d/f represent the ventrolateral wall of the LV. Scale bars: 200 μm (low magnification), 30 μm (high magnification). (**E**) Quantification of Sox2^+^ Ki67^+^ cells normalized to SVZ length. *n* = 5 independent experiments, one-way ANOVA, * *p* < 0.05, *** *p* < 0.001. Data are presented as mean ± SD.

Adult neurogenesis primarily occurs in two discrete niches: the SGZ of the hippocampal DG and the SVZ of the lateral ventricles. To explore the effect of BDNF-HA gel on adult neurogenesis in the SVZ, we first evaluated its regulatory role in NSC proliferation. BrdU was administered consecutively for 3 days to 5-month-old mice, and brain tissues were harvested 24 h after the last injection (Figure 8A). Quantification of Sox2^+^BrdU^+^ double-positive cells showed a marked decrease in proliferating NSCs in 5×FAD + PBS mice relative to WT mice, suggesting that impaired neurogenesis is present in the SVZ of 5-month-old 5×FAD mice (*p* < 0.001). Importantly, intracerebroventricular delivery of BDNF-HA gel significantly rescued this deficit, increasing the population of proliferating Sox2^+^/BrdU^+^ NSCs relative to the 5×FAD + PBS group (*p* < 0.05) (Figure 8B,C). To verify this result, we further performed Sox2 and Ki67 immunofluorescence co-staining to evaluate NSC proliferation in the SVZ (Figure 8D). Consistent with BrdU labeling results, the density of Sox2^+^Ki67^+^ proliferating NSCs was dramatically diminished in 5×FAD mice, whereas two months of BDNF-HA gel treatment markedly restored proliferative capacity (*p* < 0.05 vs. 5×FAD + PBS) (Figure 8E). Together, these results showed that BDNF-HA gel not only promotes neurosphere proliferation in vitro but also effectively stimulates endogenous NSC proliferation in the SVZ of 5×FAD mice.

### 3.10. BDNF-HA Gel Enhances Neurogenesis in the SVZ of 5×FAD Mice

**Figure 9:**
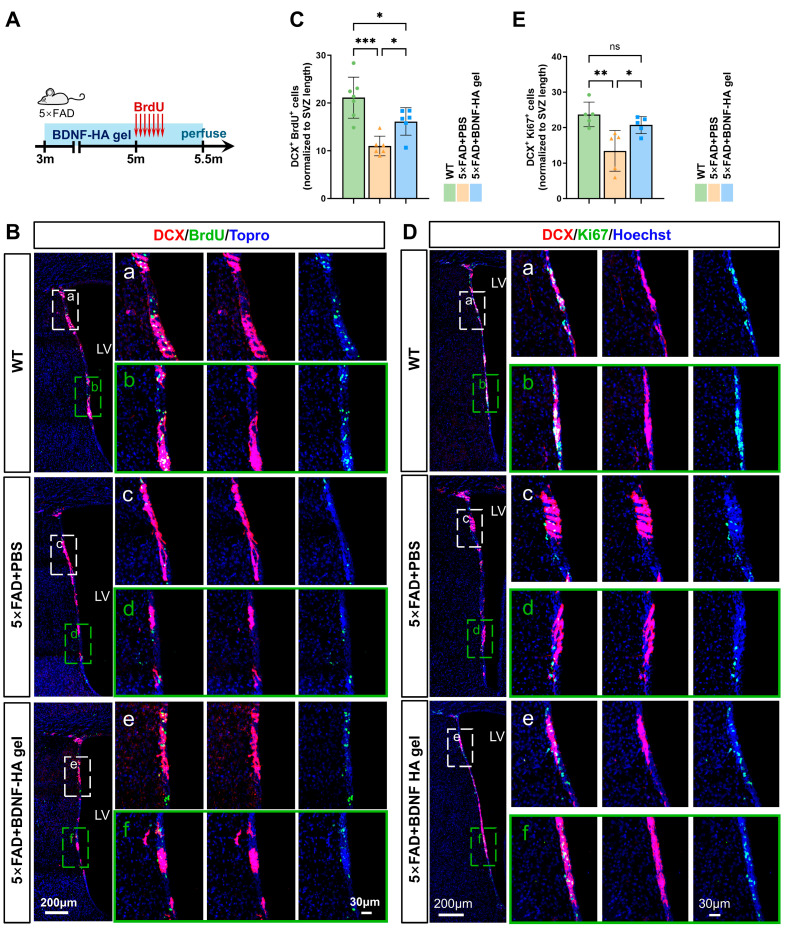
BDNF-HA gel promotes neurogenesis in the SVZ of 5×FAD mice. (**A**) Experimental timeline showing BDNF-HA gel implantation in 3-month-old mice, BrdU administration at 5 months of age, and perfusion at 5.5 months of age. (**B**) Immunofluorescence staining for DCX^+^ neuroblasts (red) and BrdU^+^ proliferating cells (green), with Topro nuclear counterstaining (blue). a/c/e represent the dorsolateral wall of the LV, while b/d/f represent the ventrolateral wall of the LV. Scale bars: 200 μm (low magnification), 30 μm (high magnification). (**C**) Quantification of DCX^+^BrdU^+^ cells normalized to SVZ length. *n* = 6~7 independent experiments, one-way ANOVA, * *p* < 0.05, *** *p* < 0.001. Data are presented as mean ± SD. (**D**) Immunofluorescence staining for DCX^+^ neuroblasts (red) and Ki67^+^ proliferating cells (green), with Hoechst nuclear counterstaining (blue). a/c/e represent the dorsolateral wall of the LV, while b/d/f represent the ventrolateral wall of the LV. Scale bars: 200 μm (low magnification), 30 μm (high magnification). (**E**) Quantification of DCX^+^ Ki67^+^ cells normalized to SVZ length. *n* = 5 independent experiments, one-way ANOVA, * *p* < 0.05, ** *p* < 0.01. Data are presented as mean ± SD. ns indicates no statistically significant difference between the two groups.

To further explore the effect of BDNF-HA gel on adult neurogenesis in the SVZ, the proliferation of neuroblasts was assessed. BrdU was administered consecutively for 7 days to mice at 5 months of age, and brain tissues were harvested at 5.5 months of age to assess the proliferation of SVZ neuroblasts (Figure 9A). Proliferating neuroblasts were identified by co-localization of BrdU with the neuroblast marker DCX (Figure 9B). Quantitative analysis revealed that compared with the WT group, the number of DCX^+^BrdU^+^ cells in the 5×FAD + PBS group was significantly decreased (*p* < 0.001). Notably, 2.5 months intervention with BDNF-HA gel markedly reversed this reduction, significantly promoting the proliferation of neuroblasts in the SVZ of 5×FAD mice (*p* < 0.05) (Figure 9C). This finding was further confirmed by DCX and Ki67 co-immunostaining (Figure 9D). Consistent with BrdU labeling, the density of DCX^+^Ki67^+^ proliferating neuroblasts was severely decreased in 5×FAD mice, whereas BDNF-HA gel administration effectively reversed this deficit (*p* < 0.05 vs. 5×FAD + PBS) (Figure 9E). Taken together, these findings demonstrate that BDNF-HA gel can effectively enhance adult neurogenesis in the SVZ of 5×FAD mice by promoting neuroblast proliferation.

### 3.11. BDNF-HA Gel Enhances Adult Hippocampal Neurogenesis in 5×FAD Mice

**Figure 10:**
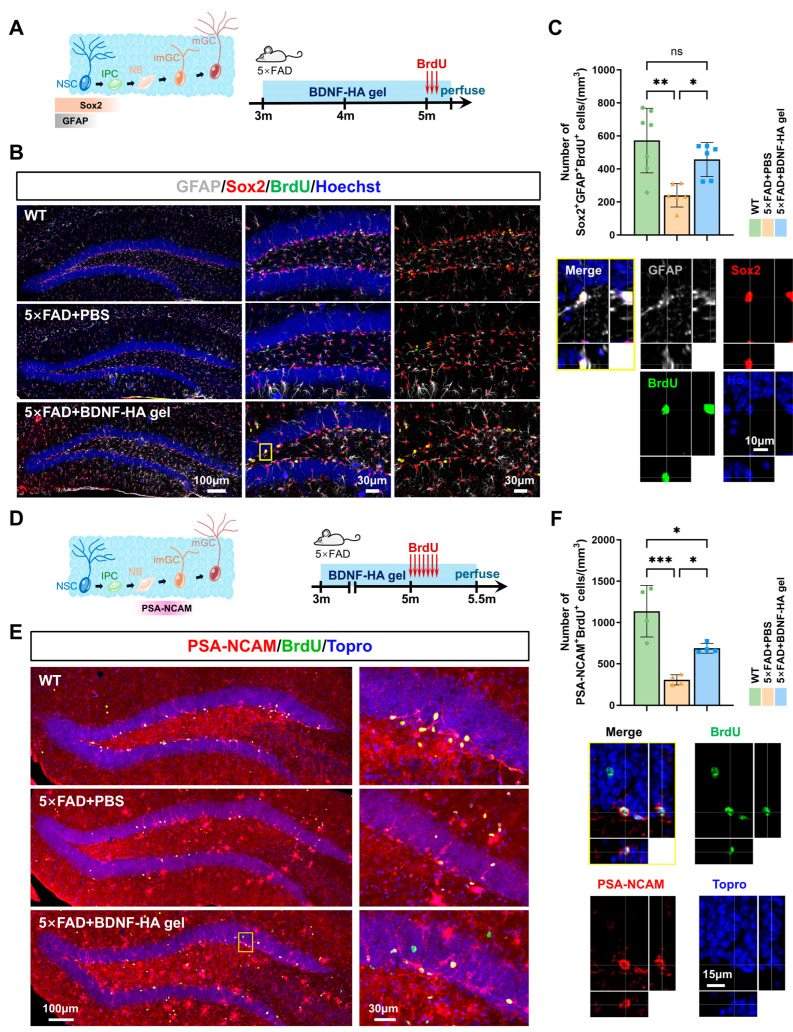
BDNF-HA gel promotes neurogenesis in the SGZ of 5×FAD mice. (**A**) Experimental timeline showing BDNF-HA gel implantation at 3 months old, BrdU administration at 5 months old, and tissue collection 24 h after the last injection. (**B**) Immunofluorescence staining for Sox2^+^ (red), GFAP^+^ (white) and BrdU^+^ proliferating cells (green), with Hoechst nuclear counterstaining (blue). The yellow box represents GFAP^+^Sox2^+^BrdU^+^ cells, and on the right is an enlarged optical section of the cells within the yellow box. Scale bars: 100 μm (low magnification), 30 μm (high magnification). (**C**) Quantification of Sox2^+^GFAP^+^BrdU^+^ triple-positive cells. *n* = 6~7 independent experiments, one-way ANOVA, * *p* < 0.05, ** *p* < 0.01. Data are presented as mean ± SD. ns indicates no statistically significant difference between the two groups. (**D**) Experimental timeline showing BDNF-HA gel implantation at 3 months old, BrdU administration at 5 months old, and perfusion at 5.5 months old. (**E**) Immunofluorescence staining for PSA-NCAM^+^ neuroblasts (red) and BrdU^+^ proliferating cells (green), with Topro nuclear counterstaining (blue). The yellow box represents PSA-NCAM^+^BrdU^+^ cells, and on the right is an enlarged optical section of the cells within the yellow box. Scale bars: 100 μm (low magnification), 30 μm (high magnification). (**F**) Quantification of PSA-NCAM^+^BrdU^+^ cells. *n* = 4 independent experiments, one-way ANOVA, * *p* < 0.05, *** *p* < 0.001. Data are presented as mean ± SD.

Beyond the SVZ, the effect of BDNF-HA gel on AHN was further investigated. To evaluate the overall proliferative activity within the entire NSC pool, Sox2^+^GFAP^+^BrdU^+^ triple-positive cells were examined. BrdU was administered consecutively for 3 days to mice at 5 months of age, and brain tissues were collected 24 h after the last injection (Figure 10A). Quantitative results showed that BDNF-HA gel treatment significantly increased the density of Sox2^+^GFAP^+^BrdU^+^ cells in 5×FAD mice (5×FAD + PBS: 240.4 ± 70.80 cells/mm^3^ vs. 5×FAD + BDNF-HA gel: 456.9 ± 103 cells/mm^3^; *p* < 0.05) (Figure 10B,C), indicating that under BDNF-HA gel intervention, activated NSCs retain their NSC identity (Sox2^+^GFAP^+^).

In addition, BrdU was administered consecutively for 7 days to mice at 5 months of age, and brain tissues were collected at 5.5 months of age to evaluate the number of proliferating neuroblasts labeled as PSA-NCAM^+^BrdU^+^ double-positive cells (Figure 10D). Quantitative analysis of immunofluorescence staining showed that compared with the WT group, the number of PSA-NCAM^+^BrdU^+^ proliferating neuroblasts in the hippocampal DG of the 5×FAD + PBS group was significantly reduced (*p* < 0.001), indicating impaired AHN in 5×FAD mice. Remarkably, 2.5 months of BDNF-HA gel treatment restored neuroblast proliferation in the 5×FAD model, as evidenced by more PSA-NCAM^+^BrdU^+^ cells compared to the PBS-treated group. (*p* < 0.05 vs. 5×FAD + PBS) (Figure 10E,F). These findings demonstrate that BDNF-HA gel effectively promotes AHN in 5×FAD mice by enhancing the survival and maturation of newborn neuroblasts in the DG.

### 3.12. BDNF-HA Gel Increases Production and Migration of Newborn Neurons in the Hippocampus of 5×FAD Mice

**Figure 11:**
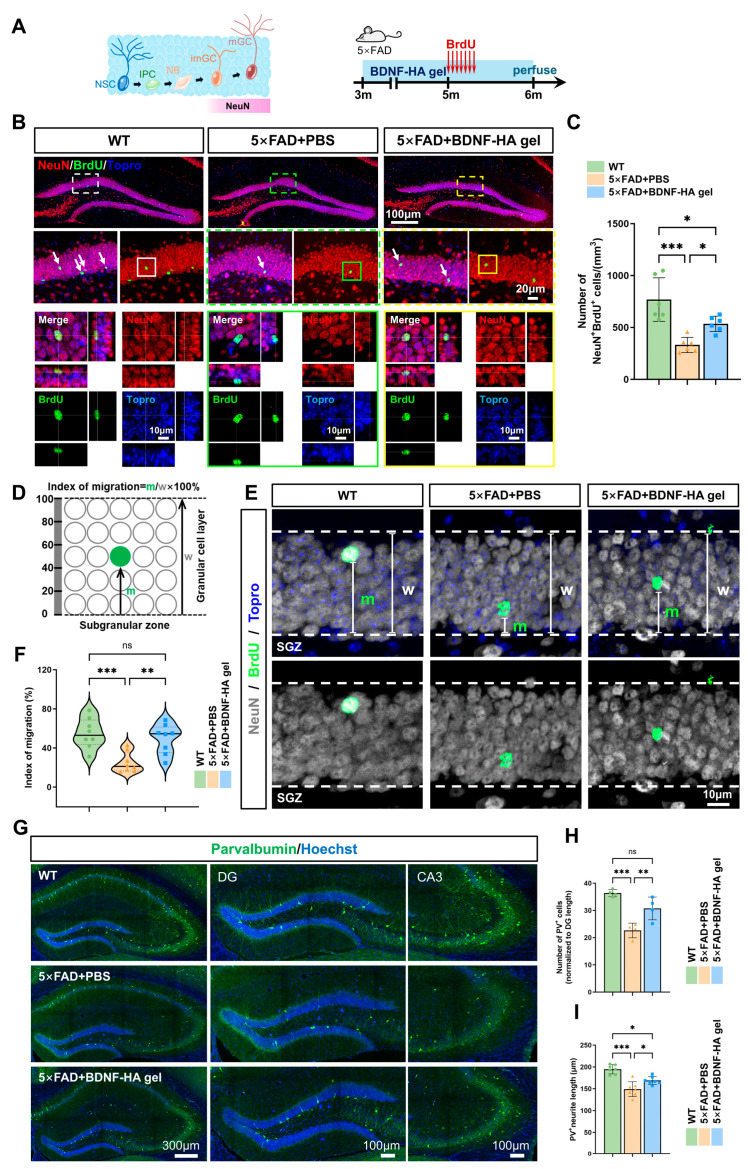
BDNF-HA gel increases the production of newborn neurons in the hippocampus of 5×FAD mice. (**A**) Experimental timeline showing BDNF-HA gel implantation at 3 months of age, BrdU administration at 5 months of age, and perfusion at 6 months of age. (**B**) Immunofluorescence staining for NeuN^+^ (red) and BrdU^+^ proliferating cells (green), with Topro nuclear counterstaining (blue). The images in the second row are magnified views of the dotted boxes in the first row for each group. White arrows indicate NeuN^+^BrdU^+^ cells. The images in the third row are optical sections of the cells within the solid boxes in the second row. Scale bars: 100 μm (low magnification), 20 μm (high magnification). (**C**) Quantification of NeuN^+^ BrdU^+^ newborn neurons. *n* = 6 independent experiments, one-way ANOVA, * *p* < 0.05, *** *p* < 0.001. Data are presented as mean ± SD. (**D**) Schematic diagram of granule cell migration. (**E**) Immunofluorescence staining of newborn neurons with NeuN/BrdU at 6 months of age. m represents the distance of cell migration, w represents the width of the dentate gyrus, and the dotted line outlines the boundary of the dentate gyrus. Scale bars: 10 μm. (**F**) Statistical analysis of migration index of NeuN^+^BrdU^+^ cells, *n* = 8 independent experiments, one-way ANOVA, *** *p* < 0.001, ** *p* < 0.01. Data are presented as mean ± SD. ns indicates no statistically significant difference between the two groups. (**G**) Immunofluorescence staining for PV^+^ cells (green) and Hoechst nuclear counterstaining (blue). Scale bars: 300 μm (low magnification), 100 μm (high magnification). (**H**) Quantification of PV^+^ GABAergic interneuron number, *n* = 4 independent experiments, one-way ANOVA, *** *p* < 0.001, ** *p* < 0.01. Data are presented as mean ± SD. ns indicates no statistically significant difference between the two groups. (**I**) Quantification of PV^+^ GABAergic interneuron dendritic length, *n* = 7 independent experiments, one-way ANOVA, *** *p* < 0.001, * *p* < 0.05. Data are presented as mean ± SD.

To evaluate the effects of BDNF-HA gel on the production and maturation of newborn neurons in 5×FAD mice, we administered BrdU to 5-month-old mice via consecutive injections for 7 days, followed by hippocampal tissue collection at 6 months of age (Figure 11A). Immunofluorescence analysis for NeuN and BrdU revealed a significant increase in the number of NeuN^+^BrdU^+^ cells in the DG of BDNF-HA gel-treated 5×FAD mice compared to the PBS-treated 5×FAD group (5×FAD + PBS: 331.8 ± 73.18 cells/mm^3^ vs. 5×FAD + BDNF-HA gel: 534.7 ± 74.18 cells/mm^3^; *p* < 0.05) (Figure 11B,C), indicating that BDNF-HA gel effectively promotes the production of newborn neurons in 5×FAD mice.

Furthermore, the migratory capacity of granular cells from the SGZ to the granular cell layer (GCL) serves as a critical indicator for evaluating neuronal development and functional integration. Specifically, the distance of granular cells within the SGZ (m) was divided by the total width of the GCL (w) at that point, expressed as a percentage: Migration Index = (m/w) × 100% (Figure 11D). Statistical results showed that the migration index of newborn neurons in the 5×FAD + BDNF-HA gel group was markedly elevated relative to the 5×FAD + PBS group (5×FAD + PBS: 24.80 ± 18.01% vs. 5×FAD + BDNF-HA gel: 41.99 ± 17.37%; *p* < 0.05) (Figure 11E,F). This finding suggests that BDNF-HA gel treatment enhances the migration of newborn neurons toward the GCL, facilitating their functional integration into the hippocampal neural circuitry.

In addition, we also examined the impact on parvalbumin-positive (PV^+^) GABAergic interneurons, which play critical roles in maintaining excitatory–inhibitory balance. Immunofluorescence staining for PV was performed on hippocampal sections (Figure 11G). Quantitative analysis revealed a significant reduction in the density of PV^+^ interneurons in the hippocampus of PBS-treated 5×FAD mice compared to WT group (*p* < 0.001). Notably, this loss was significantly attenuated in 5×FAD mice that received the BDNF-HA gel (*p* < 0.01) (Figure 11H). Morphological analysis of the surviving PV^+^ cells showed that the complexity of their dendritic arbors, assessed by measuring total neurite length, was compromised in the 5×FAD + PBS group (WT: 194.6 ± 10.53 μm vs. 5×FAD + PBS: 149.1 ± 17.00 μm; *p* < 0.001). Neurite length was significantly restored in the BDNF-HA gel-treated group, which exhibited longer neurite lengths compared to the untreated 5×FAD mice (5×FAD + PBS: 149.1 ± 17.00 μm vs. 5×FAD + BDNF-HA gel: 169.0 ± 8.92 μm; *p* < 0.05) (Figure 11I). These results demonstrate that BDNF-HA gel exerts protective effects not only on neurogenesis in glutamatergic granule cells but also on the structural integrity of GABAergic inhibitory interneurons, suggesting a broader neuroprotective role in maintaining hippocampal homeostasis.

### 3.13. The Newborn Neurons Treated with BDNF-HA Gel Anatomically Integrated into the Entorhinal–Dentate Memory Circuit

**Figure 12:**
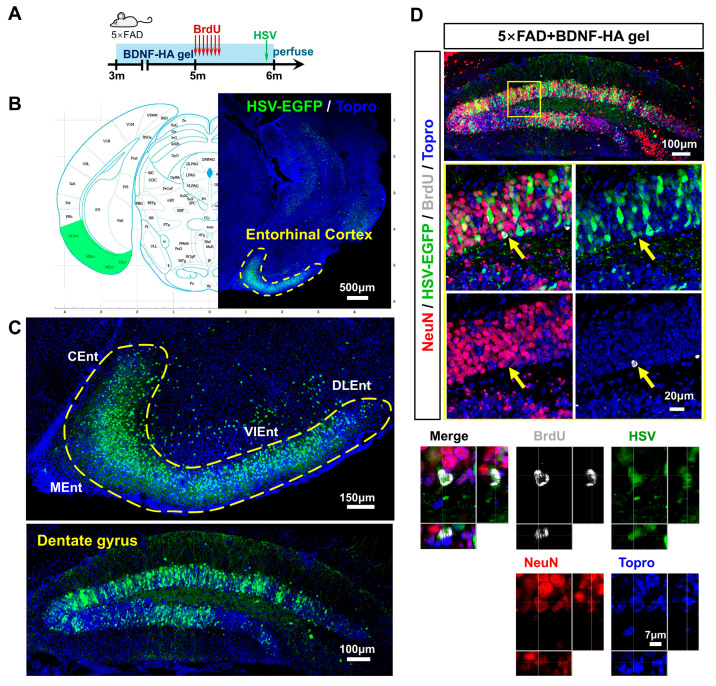
Newborn neurons generated following BDNF-HA gel treatment are anatomically integrated into the memory circuit. (**A**) Schematic diagram of the experimental procedure. (**B**) Schematic diagram showing the injection site in the EC. Both the green area on the left and the yellow dotted area on the right represent the EC. Scale bars: 500 μm. (**C**) Schematic diagram showing successful HSV infection of neurons in the EC and hippocampal DG. The yellow dotted line outlines the boundary of the EC. Scale bars: 150 μm and 100 μm. (**D**) Immunofluorescence staining for NeuN, HSV-EGFP and BrdU. The images in the second and third rows are magnified views of the yellow box in the first row. The yellow arrows point to NeuN^+^BrdU^+^HSV^+^ cells. Scale bars: 100 μm (low magnification), 20 μm (high magnification).

To investigate whether the newborn neurons treated with BDNF-HA gel achieve anatomical integration into the entorhinal–dentate (EC-DG) memory circuit, we first performed a BrdU labeling combined with anterograde transsynaptic tracing assay. At 5 months of age, 5×FAD mice were injected with BrdU for 7 consecutive days. Twenty-five days after the first BrdU injection, an anterograde trans-multisynaptic herpes simplex virus (HSV-EGFP) was injected into the entorhinal cortex (EC)—the core input region of the hippocampal memory circuit (Figure 12A). Coronal brain sections confirmed successful infection of EC neurons and subsequent trans-synaptic labeling of granule cells in the dentate gyrus (Figure 12B,C). Importantly, in the BDNF-HA gel-treated group, a subset of newborn neurons (NeuN^+^BrdU^+^ cells) was co-labeled with HSV-EGFP (Figure 12D), demonstrating that these treatment-generated neurons received direct synaptic input from the EC and were anatomically incorporated into the memory-related EC-DG circuit.

### 3.14. BDNF-HA Gel Ameliorates Cognitive Dysfunction in 5×FAD Mice

To evaluate whether BDNF-HA gel intervention can rescue cognitive dysfunction in 5.5- to 6-month-old 5×FAD mice, this study employed a comprehensive battery of behavioral tests to precisely assess different dimensions of learning and memory function in the animals, including the novel object recognition test, Y-maze spontaneous alternation test, and Barnes maze spatial reference memory test.

**Figure 13:**
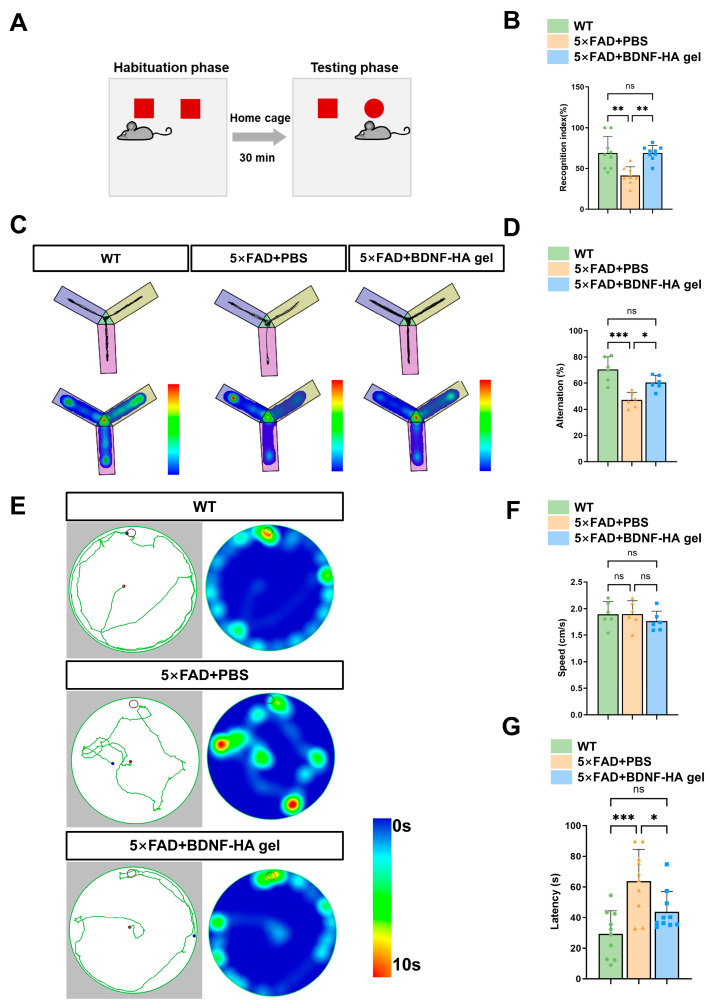
BDNF-HA gel ameliorates cognitive dysfunction in 5×FAD mice. (**A**) Schematic diagram of the novel object recognition test. Red squares represent old objects, and red circles represent new objects. (**B**) Statistical analysis of the cognitive index, *n* = 9 independent experiments. one-way ANOVA, ** *p* < 0.01. Data are presented as mean ± SD. ns indicates no statistically significant difference between the two groups. (**C**) Trajectory maps and heatmaps of the Y-maze test. (**D**) Statistical analysis of the percentage of spontaneous alternation. *n* = 6 independent experiments, one-way ANOVA, *** *p* < 0.001, * *p* < 0.05. Data are presented as mean ± SD. ns indicates no statistically significant difference between the two groups. (**E**) Trajectory maps and heatmaps of the Barnes maze test. (**F**) Statistical analysis of locomotion speed, *n* = 6 independent experiments, one-way ANOVA, No significant difference was observed among the three groups. (**G**) Statistical analysis of latency. *n* = 10 independent experiments, one-way ANOVA, *** *p* < 0.001, * *p* < 0.05. ns indicates no statistically significant difference between the two groups.

The novel object recognition test is based on the biological principle that rodents have a natural exploratory preference for novel objects—healthy normal mice tend to spend more time exploring a novel object than a familiar one (Figure 13A). The cognitive index was calculated as the ratio of the number of explorations of the novel object to the total number of explorations. This index objectively reflects the animal’s ability to retain object recognition memory. A higher cognitive index indicates stronger object recognition memory. The novel object recognition test revealed that the cognitive index of the BDNF-HA gel group was significantly higher than that of the PBS group (*p* < 0.01), suggesting that BDNF-HA gel effectively improves object recognition memory in 5×FAD mice (Figure 13B).

The Y-maze spontaneous alternation test was used to evaluate spatial working memory (Figure 13C). This test relies on rodents’ natural tendency to explore novel environments. The spontaneous alternation rate was used as the evaluation index: the percentage of consecutive entries into three different arms relative to the maximum possible alternations (total arm entries minus 2). Healthy animals with intact hippocampal-dependent spatial working memory typically exhibit a high spontaneous alternation rate, reflecting their preference for exploring novel arms. The Y-maze results showed that the spontaneous alternation percentage in the BDNF-HA gel intervention group was significantly higher than that in the PBS control group (*p* < 0.05), indicating that BDNF-HA gel intervention significantly improves hippocampal-dependent spatial working memory in 5×FAD mice (Figure 13D).

The Barnes maze test was used to assess spatial reference memory, with the primary measure being the latency to find the target hole (Figure 13E). Additionally, the search speed of the mice was recorded to exclude potential interference from motor function on memory test results. The results showed no significant difference in search speed among the test groups (*p* > 0.05), indicating that none of the animals had motor dysfunction, thus ruling out the influence of motor ability on the behavioral memory outcomes (Figure 13F). In contrast, compared with the PBS control group, the BDNF-HA gel intervention group exhibited a significantly shortened latency to find the target hole (*p* < 0.05), demonstrating that BDNF-HA gel intervention significantly improves spatial reference memory in 5×FAD mice (Figure 13G).

In summary, the consistent results from the series of behavioral tests indicate that BDNF-HA gel significantly ameliorates multiple cognitive dysfunctions in 5×FAD mice, including object recognition memory, spatial working memory, and spatial reference memory.

## 4. Discussion

In this study, NSC spheres were successfully isolated from the hippocampal tissues of neonatal rats. To mimic the impact of Aβ on NSCs in the AD pathological microenvironment, the culture medium was supplemented with Aβ_1–42_ oligomers to establish an in vitro model. This experimental system successfully replicated key pathological hallmarks of AD, including neurosphere apoptosis and reduced NSC proliferation, consistent with the impaired neurogenesis observed in AD animals [23]. The Aβ_1–42_ oligomer-induced model offers several advantages: (1) it enables precise manipulation of both Aβ concentration and exposure time, facilitating detailed dose–response and temporal analyses of NSC behavior [24]; (2) it provides a homogeneous and reproducible experimental system that eliminates confounding factors present in the complex in vivo environment; (3) it facilitates direct observation of cell-autonomous effects of Aβ toxicity on NSC fate determination without systemic interference; and (4) it serves as a simple and efficient platform for screening neuroprotective interventions [25]. These properties make the model suitable for testing therapies against Aβ neurotoxicity. Using this system, Aβ_1–42_ oligomers suppressed NSC proliferation and inhibit the entire neurogenic cascade, while BDNF-HA gel delivery significantly mitigated these inhibitory effects. Moreover, Aβ toxicity reduced the differentiation of NSCs into Tuj1^+^ neurons and Olig2^+^ oligodendrocytes, which was effectively counteracted by BDNF-HA gel treatment. Further exploration revealed that BDNF-HA gel could mitigate Aβ_1–42_-induced mitochondrial dysfunction. Given the close association between mitochondrial dynamics and neurogenesis [26,27,28], this protective effect on mitochondria likely constitutes one of the vital mechanisms through which BDNF-HA gel promotes neurogenesis. Importantly, preliminary investigations in 5×FAD mice confirmed the in vitro results. BDNF-HA gel boosted neurogenesis in both the SVZ and DG despite persistent Aβ plaque pathology. BDNF-HA gel sustains neurogenesis across diverse Aβ toxicities—from oligomers to plaques.

Neurotrophins are indispensable for repairing central nervous system injuries, and numerous studies have confirmed their efficacy in treating cognitive decline [29,30,31,32]. Neurotrophin depletion during aging and neurodegeneration accelerates neuronal injury and cognitive collapse [33]. Neurotrophic factors associated with AD mainly include NGF and BDNF [29,34]. AD patients exhibit impaired NGF metabolism [35], characterized by elevated levels of the precursor proNGF and decreased mature NGF in brain tissues of subjects with both AD and MCI [36]. This phenomenon is mainly caused by the suppressed activity of tissue plasminogen activator (tPA) and elevated neuroserpin levels in AD brains, both of which block proNGF conversion to mature NGF. Accumulated proNGF prefers p75NTR-mediated pro-apoptotic signaling, promoting neuronal degeneration [37,38,39]. Likewise, impaired BDNF signaling has been widely reported in AD. Diminished TrkB activation and imbalanced proBDNF/mature BDNF ratios are closely associated with synaptic degeneration and cognitive impairment [8,40]. Exogenous BDNF supplementation thus serves as a promising therapeutic strategy to restore cognitive function in AD. However, the direct administration of BDNF is often ineffective due to its short half-life, rapid degradation, and poor ability to cross the blood–brain barrier [41]. Currently, numerous investigators have utilized lentiviral-mediated BDNF overexpression [7]. However, this strategy presents limitations for clinical translation.

To overcome these limitations, nanomaterial-based delivery systems have been developed to improve BDNF stability and extend its local retention, thereby achieving sustained BDNF release and prolonged biological activity. For instance, Fang et al. utilized Fe_3_O_4_-based nanoparticles to deliver BDNF in AD rats, which significantly enhanced memory and cognitive functions [42]. Similarly, another study employed amino-acid–catecholamine hybrid nanoparticles for BDNF loading, also achieving effective restoration of memory function [43]. Nanocarriers thus solve BDNF’s delivery problems. Accordingly, HA hydrogel was selected to encapsulate BDNF in the present study. When applied to the in vitro AD model induced by Aβ_1–42_ oligomers in NSCs, BDNF-HA gel was found to promote neurite outgrowth of NSCs and inhibit apoptosis of NSC spheres. These findings demonstrate that the BDNF-HA gel can effectively preserve the neuroprotective functions of BDNF, highlighting its potential as a therapeutic strategy for mitigating Aβ-induced damage and supporting neuroregeneration. Although encapsulated BDNF with nanomaterials can significantly increase BDNF expression in vivo, tail vein injection and intracerebroventricular injection each has certain limitations for clinical translation. These routes of administration are feasible in experimental animals, but they result in poor long-term treatment compliance for AD patients. Therefore, it is necessary to explore more convenient and effective alternative routes of administration.

Beyond its neuroprotective effects, BDNF is more importantly associated with neurogenesis. Multiple studies have confirmed that elevating BDNF levels holds promise for treating neurogenesis impairment in AD, thereby improving memory function [7,16,17,18,19]. Our study found that BDNF encapsulated in HA hydrogel promotes neurogenesis in NSCs damaged by Aβ_1–42_ oligomers, as evidenced by increasing NSC proliferation, facilitating the differentiation into neuroblasts and newborn neurons. BDNF mainly exerts its effects by binding to and activating the high-affinity TrkB receptor, which plays a key role in neurogenesis [44,45,46]. Similarly, Charou et al. systematically evaluated a novel small-molecule TrkB agonist, ENT-A011. In mouse and human neural models, ENT-A011 proved to be a selective TrkB agonist. It promoted NSC proliferation and differentiation, enhanced survival, and blocked Aβ neurotoxicity [25]. ENT-A011 activated TrkB as strongly as BDNF. RNA sequencing further revealed that ENT-A011 shares a core gene regulatory network with BDNF, yet ENT-A011 modulates more targets and persists longer [25]. These results demonstrate that sustained, slow release of BDNF, via binding to TrkB receptors, can promote neurogenesis. The BDNF-HA sustained-release system employed in our study offers an alternative and promising platform for AD treatment strategies.

Neurogenesis is an energy-intensive process that encompasses asymmetric division, differentiation, migration, and synaptic integration of NSCs [47], all of which are critically dependent on robust mitochondrial function [48]. Mitochondria also serve as key organelles regulating the fate determination, proliferation, differentiation and survival of NSCs [49]. Mitochondrial dysfunction occurs prior to the formation of Aβ plaques and neurofibrillary tangles in AD and represents an early biomarker of the disease [50,51]. For instance, in AD transgenic mouse models, mitochondrial impairment manifests months before the onset of cognitive symptoms [52]. Similarly, postmortem brain tissues from patients with MCI already exhibit significant defects in mitophagy [53]. Consistent with these findings, Zhao et al. demonstrated that treating primary neurons with 10 μM Aβ_1–42_ for 24 h to model AD pathology led to the collapse of MMP [54]. These findings are consistent with our results showing a decrease in MMP following Aβ_1–42_-induced injury. It is widely recognized that Aβ pathology also impairs NSC viability by disrupting mitochondrial function [55]. Mitochondrial dysfunction inhibits AHN. Aβ irreversibly disrupts mitochondrial biogenesis, dynamics, and redox pathways in self-renewing NSCs, impairing neurogenic differentiation [55]. Therefore, restoration of mitochondrial function emerges as a critical determinant for ensuring neurogenesis in the context of Aβ pathology. Like BDNF, ENT-A011, as a novel BDNF mimetic and a TrkB agonist, significantly reverses Aβ-induced mitochondrial dysfunction via TrkB receptor activation. This suggests that preserving mitochondrial function may be central to its neurogenic activity [25]. Similarly, the present study found that BDNF-HA gel improves mitochondrial function, which is crucial for promoting neurogenesis.

Several limitations of this study remain to be addressed in future investigations. First, the activation of the BDNF-TrkB axis and its downstream signaling cascades was not systematically investigated. Second, the BDNF dosage requires further optimization. Inadequate BDNF may fail to effectively promote adult neurogenesis, while excessive BDNF may exert neurotoxic effects. Third, although anatomical integration of newborn neurons into the entorhinal–dentate memory circuit was demonstrated, functional validation remains to be performed. Finally, BDNF exerts synaptoprotective effects and synaptic integrity is closely linked to cognitive function. Therefore, future studies should examine synaptic structure and function.

## 5. Conclusions

In summary, BDNF-HA gel promotes NSC proliferation, migration, neuronal differentiation, and mitochondrial function under Aβ-toxic conditions. In 5×FAD mice, it enhances adult neurogenesis in the SVZ and DG, facilitates anatomical integration of newborn neurons into the EC-DG memory circuit, and ameliorates cognitive deficits without reducing Aβ burden. These results demonstrate that sustained BDNF delivery via HA gel effectively restores endogenous neurogenic capacity, offering a promising amyloid-independent regenerative strategy for AD.

## Data Availability

The original contributions presented in this study are included in the article. Further inquiries can be directed to the corresponding author.

## Abbreviations

The following abbreviations are used in this manuscript:

AD	Alzheimer’s disease
AHN	adult hippocampal neurogenesis
NSCs	neural stem cells
BDNF	brain-derived neurotrophic factor
HA	hyaluronic acid
SVZ	subventricular zone
SGZ	subgranular zone
DG	dentate gyrus
BrdU	5-Bromo-2′-deoxyuridine
APP	amyloid precursor protein
PSEN1	presenilin 1
PSA-NCAM	polysialylated neural cell adhesion molecule
